# An assessment of the health system’s capacity for major structural birth defects care in Tanzania using anorectal malformations as a tracer condition: A multi-phase, mixed-methods study protocol

**DOI:** 10.1371/journal.pone.0359639

**Published:** 2026-10-05

**Authors:** Masawa K. Nyamuryekung’e, Kokila Lakhoo, Salome Maswime

**Affiliations:** 1 Department of Surgery, Aga Khan University Medical College, Dar es Salaam, Tanzania; 2 Nuffield Department of Surgical Sciences, University of Oxford, Oxford, United Kingdom; 3 Global Surgery Division, Department of Surgery, University of Cape Town, Cape Town, South Africa; University of Waterloo, CANADA

## Abstract

**Background:**

Structural birth defects cause disproportionate neonatal and under-five morbidity and mortality in low- and middle-income countries, where 90–95% of births and associated deaths occur; however, health-system readiness for prevention uptake, early detection, and management remains poorly understood. Anorectal malformations account for 33–40% of the paediatric surgical workload in sub-Saharan Africa, making it a tracer condition for evaluating Tanzania’s capacity across primary, secondary, and tertiary prevention levels. Although delayed detection, high postoperative morbidity, and referral dysfunction are documented, the upstream preconception, antenatal, delivery, and postnatal profile, parental lived experience, external benchmarking of paediatric surgical capacity, and feasibility of a context-specific preoperative checklist remain to be quantified within the Tanzanian clinical environment.

**Methods:**

This multi-phase mixed-methods protocol comprises four phases. In Phase I, all mother-newborn dyads where the infant has an anorectal malformation presenting to Muhimbili National Hospital will be enrolled in a cross-sectional study quantifying preconception, antenatal, delivery, and postnatal prevention uptake; multivariable logistic regression will identify predictors of early detection; the sample size will be derived via Cochran’s formula using finite population correction. Phase II will use descriptive phenomenology through purposive in-depth interviews with parents exploring socio-cultural access determinants, analysed via the Stevick-Colaizzi-Keen method. Phase III will conduct a facility-level survey against the Optimal Resources for Children’s Surgery checklist at four tertiary hospitals; domain-specific and total compliance scores will be calculated, gaps quantified against benchmarks, and centres compared using Friedman tests and chi-square or Fisher’s exact tests. Phase IV will test feasibility of a preoperative anorectal malformation checklist at Muhimbili National Hospital, examining practicality and acceptability (non-parametric comparisons).

**Discussion:**

This study will produce a system-level appraisal of Tanzania’s capacity to prevent, detect, and manage major structural birth defects using anorectal malformation as a tracer condition. Findings will inform policy, guide resource allocation, and support targeted interventions.

## Background

Ninety to ninety-five percent of birth defects and their associated mortalities occur in Low- and Middle-Income Countries [1]. The burden is concentrated in sub-Saharan Africa, where gaps in preconception and antenatal care, and under-developed neonatal surgical and surveillance systems intersect [2–4]. In the case of anorectal malformation (ARM), like other major structural birth defects, emergency surgery averts mortality [5]. Because associated malformations are common, long-term follow-up care across the life course improve the long term outcomes [6]. These features make ARM a suitable tracer condition for assessing the health system’s capacity for major structural birth defects prevention, early detection, and management across birth defects prevention levels [7].

Internationally, ARM is one of the most commonly encountered congenital structural surgical conditions and constitutes 33–40% of the pediatric surgical workload in sub-Saharan Africa [8–10]. In well-resourced health systems, ARM is increasingly managed as a survivable neonatal condition with low operative mortality, structured follow-up care maintain lifelong quality of life [11–13]. In resources-limited health systems, clinical outcomes diverge significantly. In Tanzania neonatal ARM emergency surgery is associated with morbidity rates of 47%, reoperation rates of 16%, and mortality rates of 16%, these findings are similar across the wider sub-Saharan Africa pediatric surgical literature [8,9]. From a paradigm that recognizes surgical outcomes as a reflection of capacity of health systems, this pattern signals gaps in different disease prevention levels [14].

The World Health Organization recommends a birth defects prevention level framework for birth defects. The goals at the primary birth defects prevention level are preconception risk factors reduction and protective factors optimisation, at the secondary birth defects prevention; early detection through prenatal and postnatal screening and appropriate referral, and tertiary birth defects prevention; stabilisation, management, and long-term care. These levels are supported by a continuing call for surveillance, integrated and a continuum-of-care approach to promote an efficient sustainable birth defects prevention, early detection, and management program [2,4,7,15]. When contextualised to ARM, this framework is only partially applicable. There are no ARM-specific primary prevention interventions; the generic primary birth defects prevention entry points (teratogenic exposure minimisation, nutrition optimisation, family planning, and genetic counselling [16–20], are shared across all major structural birth defects. Despite the generic entry points at birth defects primary prevention, in sub-Saharan Africa less than one in four reproductive-aged women access preconception care, only one in ten eligible women achieve folic acid supplementation, and only 32% possess good knowledge of preconception interventions [19,20].

At the secondary birth-defects prevention level, early detection during prenatal screening is commonly achieved using antenatal ultrasound. In ARM it has limited utility for early detection [15,21]. ARM lacks distinctive prenatal ultrasonographic features that would enable reliable prenatal identification [22]. Consequently, the most feasible ARM, and other major structural birth defects early detection strategy is systematic postnatal newborn examination [21]; the timeliness of appropriate referrals determine outcomes in the emergency neonatal presentation [23]. Screening programmes for birth defects in resource limited settings are not fully established and major structural birth defects are frequently missed during the initial newborn examination [15,21,24]. For ARM specifically, more than half of affected newborns are diagnosed after 48 hours of life, a delay that substantially increases mortality risk and reflects constrained secondary birth defects prevention capacity [24]. A recent retrospective chart review study from Tanzania [25] further underscored this early detection gap, reporting delayed presentation in 77% of ARM cases; only 31.6% of ARM cases were first identified by healthcare workers, while mothers detected 68.4% [25]. This study’s exploration was limited to documented variables without access to upstream determinants across the Reproductive, Maternal, Newborn, Child and Adolescent Health continuum. The current study aims to extend understanding of the detection-gap prospectively by enrolling incident ARM mother–newborn dyads at the point of emergent presentation at a tertiary hospital in Phase I. Our hypothesis is that early detection of ARM is associated with upstream maternal and facility-level factors, specifically (i) antenatal care uptake and content, (ii) cadre and practices of birth attendants, and (iii) completeness and timing of newborn examination at the facility of birth.

Even after a child with ARM reaches the tertiary-level health facility, in-hospital delays are frequently encountered. Tertiary facilities are consistently overwhelmed due to dysfunctional referral pathways, a limited specialised workforce and infrastructure, and the absence of dedicated multidisciplinary care and in‑hospital processes required for timely emergent ARM surgery [2,26]. In Tanzania, the resulting effects of these systemic limitations is striking: after emergency surgery for ARM, 47% sustain morbidities, 16% require reoperations, and 16% sustain mortality [8], findings corroborated by broader sub-Saharan Africa paediatric surgical outcomes data [9,14,26].

These outcomes reflect gaps in neonatal surgical readiness. Definitive management for major structural birth defects, which often requires neonatal emergency surgery, is delivered at tertiary hospitals, and capacity at this level in Tanzania remains unassessed [27]. The current study assesses paediatric surgical capacity at the tertiary level using ARM as a tracer condition, benchmarks the capacity against an international standard framework [28] in phase III. To address the absence of dedicated multidisciplinary care and the in‑hospital processes required for timely emergent ARM surgery, Phase IV will conduct a feasibility study of a preoperative tool designed to reduce in‑hospital delays. Our hypothesis is that Tanzanian tertiary‑level paediatric surgical capacity will differ significantly across hospitals due to variations in infrastructure, workforce, service delivery, equipment availability, and governance.

Beyond the emergent neonatal stage, ARM management proceeds through staged surgical interventions to correct the anatomy, followed by long-term care focused on functional outcomes and quality of life [29]. Across this continuum, social, cultural, and economic circumstances profoundly shape families’ access to care and their ability to sustain the treatment pathway [30]. Stigma, abandonment, and family separation have been reported, underscoring the significance of sociocultural factors in determining access to healthcare for an ARM-affected child [31]. These factors [32] act as either barriers or facilitators to healthcare access. In Tanzania, how these factors are experienced and navigated, and how they intersect with health service delivery, remains unexplored [30,33]. This gap provides the rationale for the qualitative phenomenological phase II of this study.

### Conceptual framework for integrated structural birth defects prevention, early detection, and management

To assess a health system’s major structural birth defects prevention, early detection, and management, an appropriate conceptual framework is required. Fig 1 is a depiction of the current study’s proposed framework.

**Fig 1:**
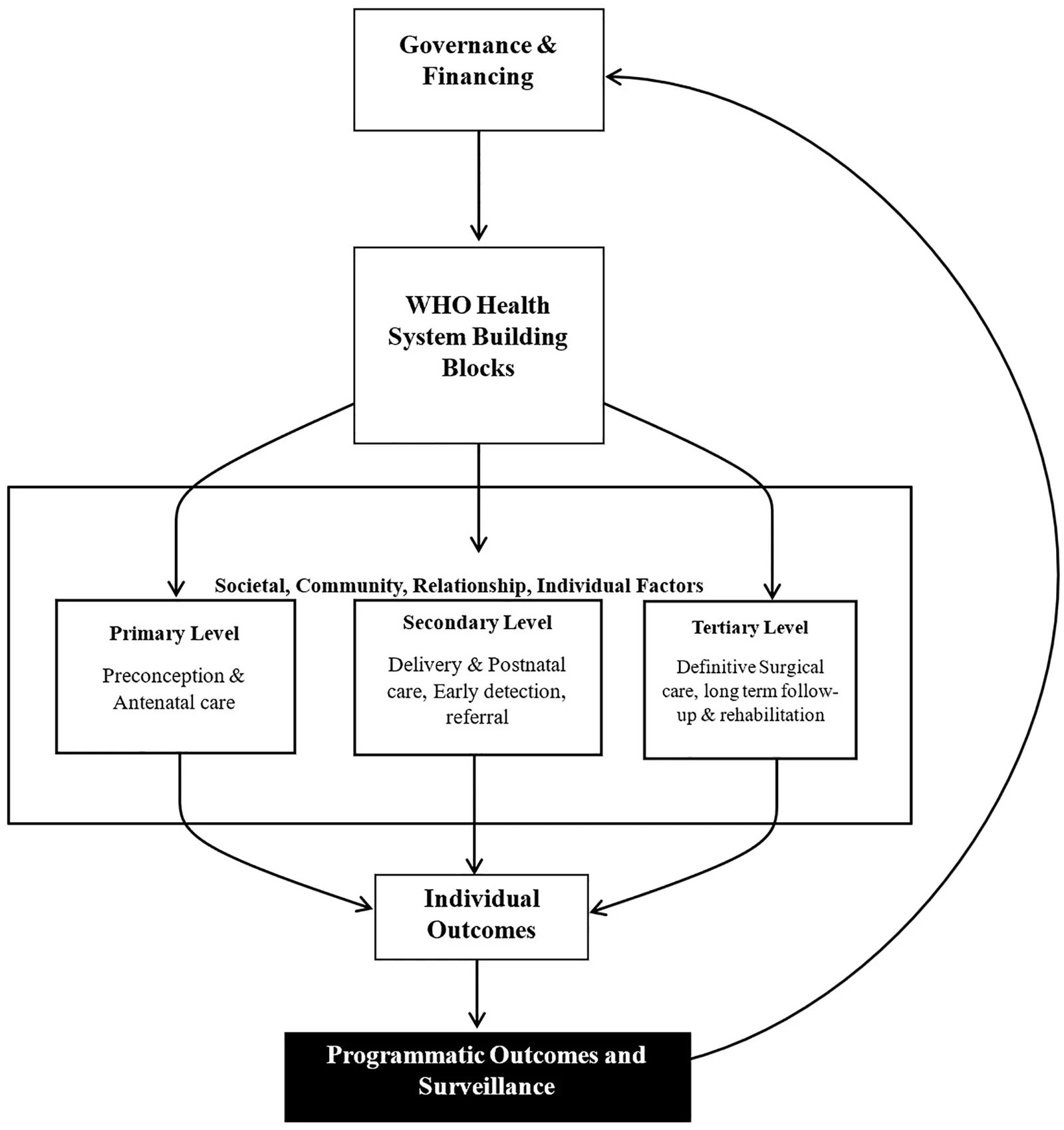
Conceptual framework for integrated structural birth defects prevention, early detection, and management. Conceptual framework linking governance and financing to WHO health‑system building blocks, levels of care (primary, secondary, tertiary), sociocultural determinants, and resulting individual and programmatic outcomes across the ARM care continuum.

In the proposed framework, the first tier (governance, financing, and planning) provides the overarching policy and resource environment for all interventions. The second tier components (service delivery, workforce, information systems, medical products, financing, and leadership) [34] are the essential enablers of care, shaped and sustained by first-tier components.

On the third tier, care delivery spans the referral system. At the primary level (preconception and antenatal care) the focus is on risk reduction, maternal health optimisation, and early birth defects and genetic counselling; uptake of these interventions is described in Phase I. At the secondary level, emphasis shifts to newborn examination, early detection, and timely referral to higher-level facilities; assessed in Phase I. At the tertiary level, urgent surgical care is provided according to available capacity, assessed in Phases III and IV [28]. Access to the third tier is shaped by societal, economic, community, relationship, and individual forces, explored in qualitative Phase II.

The impact of the above tiers culminates in individual physical, psychosocial, and functional outcomes. Collectively, the individual outcomes inform programmatic outcomes through surveillance data and reporting, creating system feedback that promotes evidence-based planning, resource allocation, and continuous improvement in the prevention, early detection, and management of major structural birth defects. This multiphase study aims to provide the first health-system-wide appraisal of Tanzania’s ability to early detect and manage major structural birth defects using ARM as a tracer condition through the lens of this conceptual framework.

### Study objectives

1. To determine factors associated with the early detection of ARM in newborns
2. To explore the sociocultural, economic, and belief-based determinants influencing healthcare access among parents of children with ARM at Muhimbili National Hospital
3. To compare the existing Tanzanian paediatric surgical care capacity against the optimal standards recommended by Optimal Resources for Children’s Surgery
4. To assess the feasibility of implementing a preoperative checklist for neonates with ARM at Muhimbili National Hospital

## Methods

### Study design, setting, and duration

A multi-phase mixed-methods design will be employed. Phases I, II, and IV will be conducted at Muhimbili National Hospital, the institutional anchor for ARM‑related components, while Phase III will extend to additional tertiary hospitals with at least one full-time pediatric surgeon to enable a national assessment of paediatric surgical capacity. Muhimbili National Hospital (MNH) has the largest paediatric surgical caseload in Tanzania. A 2022–2023 retrospective review documented 152 ARM admissions on various stages of care [25], with a consistent annual volume of 15–35 newborn cases presenting for first ARM surgical intervention. At study conception (2024), MNH (Dar es salaam) was one of only four tertiary hospitals in Tanzania with at least one full‑time paediatric surgeon. The other centres included the Aga Khan Hospital (AKH), a private tertiary facility in Dar es Salaam; Bugando Medical Centre (BMC), a faith‑based Lake Zone and western Tanzania referral hospital; and Mbeya Zonal Referral Hospital (MZRH), serving the southern highlands. Each zonal hospital serves an estimated 5–15 million people [35]. All participating centres reviewed the protocol, which included the comparative benchmarking analysis, and granted institutional approval for participation. Fig 2 illustrates the timelines, phases, and setting for this multiphase study.

**Fig 2:**
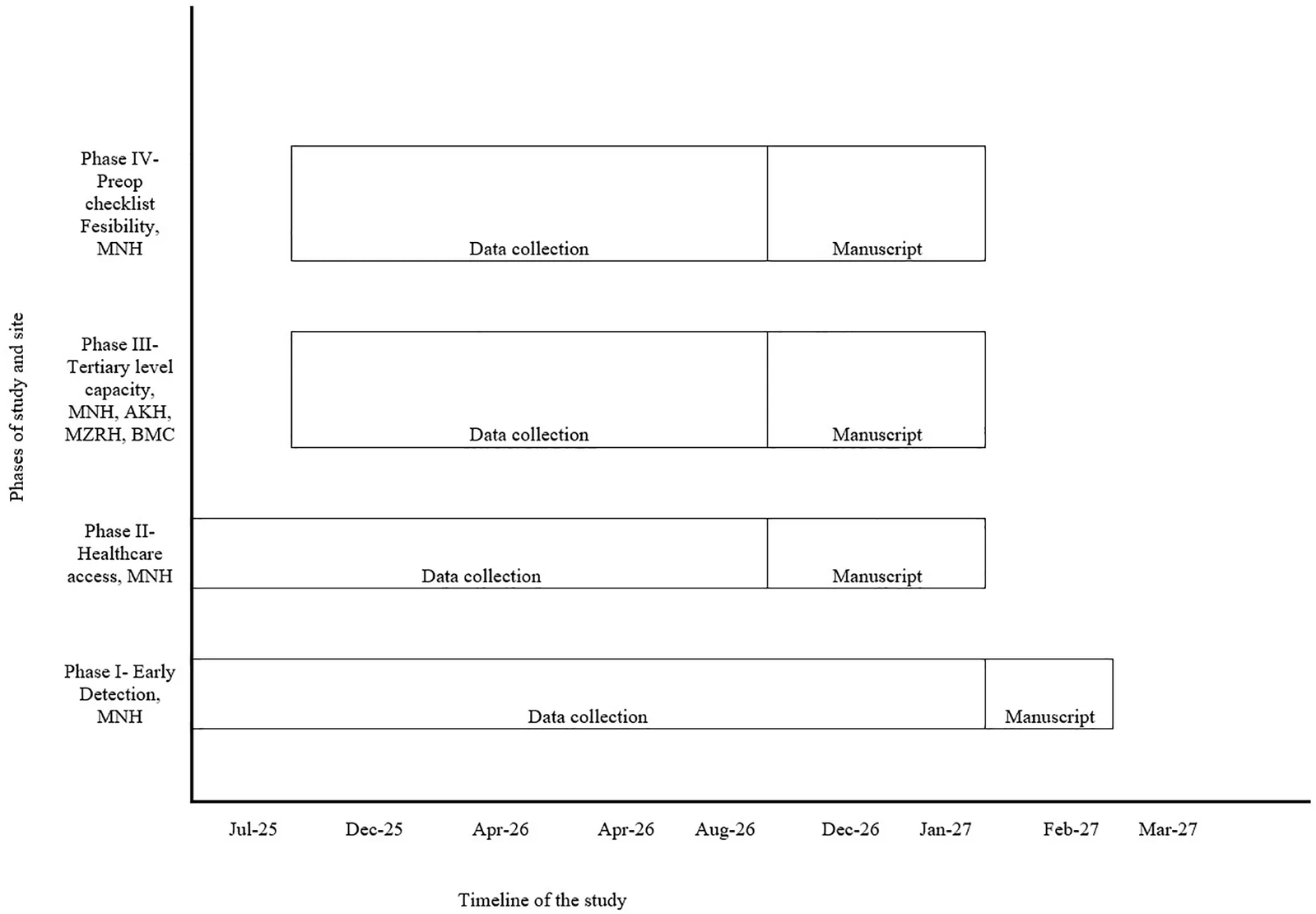
Timelines, phases and settings for the different phases of the study. Timeline of the four‑phase study, showing periods of data collection and manuscript preparation across sites (MNH, AKH, MZR, BMC) from July 2025 to March 2027. Each phase corresponds to a distinct component of the research: early detection (Phase I), healthcare access (Phase II), tertiary-level capacity assessment (Phase III), and preoperative checklist feasibility (Phase IV).

### Phase I

#### Study design.

Phase I will employ a prospective cross-sectional analytical design

#### Study population, inclusion and exclusion criteria.

Primary caregivers of newborns with ARM presenting for the first surgical intervention will be included. Primary caregivers lacking the capacity for informed consent will be excluded.

#### Sample size estimation.

The standard single-proportion Cochran’s formula was utilized.


$$\begin{array}{c} n=\frac{z^{\text{2}}Xp(\text{1}-p)}{e^{\text{2}}} \end{array}$$

The expected early detection rate, defined as diagnosis before discharge from the facility of birth, is 50% [24]. A margin of error (e) of 5% and a confidence interval of 95%, corresponding to a z-score of 1.96, were applied.

ARM is a rare condition [5]. MNH admits about 15–35 neonates with ARM cases per year requiring the first urgent surgical intervention [36], giving an estimated 52 eligible participants over an 18‑month enrolment period. All consecutive cases will be recruited.

Using N = 52 as the finite population, the sample size derived from Cochran’s formula was adjusted using the finite population correction. A minimum of 46 participants will be required. All consecutive cases will be recruited during the enrollment window.


$$\begin{array}{c} n=\frac{n}{\text{1}+\frac{\left(n-\text{1}\right)}{N}} \end{array}$$

#### Variables.

Maternal socio‑demographic characteristics, periconception exposures, obstetric history will be captured. Regarding the index pregnancy with the ARM-affected newborn, preconception care uptake, antenatal care uptake, and delivery‑related characteristics, and prompt postnatal care characteristics and uptake will be captured. See S1 File, Phase I data collection tool. The primary outcome is early ARM detection, defined as diagnosis before discharge or within 72 hours [37]. See S1 File. Phase I Data-Collection Tool

#### Data collection tool and procedures.

The data-collection tool was developed by the research team using the Tanzanian Reproductive, Maternal, Newborn, Child and Adolescent Health continuum of care as the guiding framework [38]. Components relevant to early ARM management across preconception, antenatal, delivery, and immediate postnatal care were adapted to ensure contextual relevance and alignment with national standards [38].

Data will be collected using a structured, researcher-administered electronic questionnaire. Research assistants will receive two days of training on standardised data collection procedures. To maintain data integrity, 10% of records will undergo monthly review, with any systematic issues addressed through targeted retraining.

#### Data analysis.

Descriptive statistics using frequencies, percentages, and medians will be used. The binary outcome is early ARM detection. Distinct prespecified clinically relevant predictors [25] will include birth defects awareness before delivery, delivery facility, Birth attendant characteristics, prompt postnatal care characteristics and uptake will be assessed Fisher’s exact or chi-square tests for categorical variables, and Mann–Whitney U or Kruskal–Wallis tests for continuous or ordinal variables. Effect sizes will be reported using Cramér’s V, rank-biserial correlation (r = Z/√N), and epsilon‑squared (ε²), interpreted using established benchmarks (weak, moderate, strong) [39].

Given the rarity of ARM and the anticipated sample size, multivariable modelling will be restricted to a maximum of three prespecified clinically relevant predictors. Predictor selection will be guided by the study’s conceptual framework and existing literature rather than data-driven selection alone. The model will only be fitted if the events-per-variable criterion can be met; otherwise findings will be limited to univariable analyses with effect-size estimation. The multivariable analysis is intended to explore independent associations and generate hypotheses rather than produce definitive predictive estimates. Model assumptions will be evaluated through checks for multicollinearity (Variance Inflation Factors), linearity of continuous predictors with the logit (Box–Tidwell), and influence diagnostics using standardised residuals. Final results will include adjusted odds ratios with 95% confidence intervals, p-values, and model performance metrics, including the Hosmer–Lemeshow test and area under the ROC curve. All computational procedures will be performed in IBM SPSS Statistics version 25 (IBM Corp., Armonk, NY, USA). Reporting for this phase will adhere to Strengthening the Reporting of Observational Studies in Epidemiology guidelines [40].

### Phase II

#### Study design.

Phase II will use a qualitative descriptive phenomenological design grounded in a constructivist epistemology.

#### Study population, inclusion and exclusion criteria.

Parents of neonates with ARM at different points along the care pathway will be included. Eligibility requires that the parent be the child’s primary caregiver. Purposive sampling will maximize variation by recruiting caregivers whose children are at different stages of surgical treatment of ARM.

#### Sample size estimation.

Data collection and analysis will proceed iteratively, informing subsequent sampling. Interviews will continue until code saturation, defined as three consecutive interviews yielding no new codes [41]. Based on prior studies, it is anticipated that saturation will be reached after approximately 9–20 in-depth interviews [41].

#### Research team, reflexivity and positionality.

Parent interviews will be conducted by RAB, a male general surgeon with prior qualitative interviewing experience, and attended hands-on workshop in individual in-depth interview. To mitigate preexisting power dynamics, the interviewer will have had no prior contact with participants and informed consent will be obtained with the researcher’s role clearly explained. Open‑ended, participant‑led questioning will be used to create a comfortable environment and minimize hierarchical influence.

Reflexivity and trustworthiness will be supported through bracketing and ongoing reflexive memoing, documenting nonverbal cues, emerging assumptions, and positionality reflections. These memos will be utilized to facilitate iterative team discussions to moderate bias. To minimise bias, the interviewer (RAB) will not participate in transcript analysis. The analysis team will include Prof. KL (pediatric surgery, global pediatric surgery, United Kingdom), Prof. SM (obstetrics and gynaecology, global surgery, South Africa), and MKN (pediatric surgery, global surgery, Tanzania).

#### Data collection tool and procedures.

Individual in-depth interviews will use a guide co-developed by MKN, KL, and SM based on ARM literature, clinical expertise, and the study’s theoretical framework [32]. Following a pilot of the interview guide with one parent, minor refinements were implemented in the framing of the questions. Interviews will be conducted privately in Swahili, audio-recorded, transcribed verbatim, and translated into English; bilingual team members (MKN and RAB) will independently check selected transcripts to preserve meaning. See S2 File. Phase II Interview Guide

#### Analysis and trustworthiness.

Data will be analysed using the Modified Stevick‑Colaizzi‑Keen phenomenological [42] method to explore and describe the social, cultural, belief‑related, and economic experiences influencing healthcare access among parents of children with ARM. Analysis will follow the core SCK steps: bracketing, horizontalization, extraction of significant statements, formulation of meanings, clustering into themes, development of an exhaustive description, and synthesis of the fundamental structure of the lived experience. Analysis will be conducted in MAXQDA (version 26.3; VERBI Software, Berlin, Germany).

Trustworthiness will be ensured through credibility (reflexive memoing, attention to non‑verbal cues, iterative clarification), dependability (maintaining an audit trail), confirmability (bracketing and team‑based analysis across diverse disciplinary perspectives), and transferability (providing thick contextual descriptions). These strategies support a rigorous and phenomenologically grounded interpretation of caregiver experiences. Reporting for this phase will adhere to the Consolidated Criteria for Reporting Qualitative Research guidelines [43].

### Phase III

#### Study setting and design.

Phase III will be a facility‑based survey conducted at the four tertiary hospitals in Tanzania with at least one full‑time paediatric surgeon.

#### Survey respondents.

Respondents will include heads of the anaesthesiology, paediatric surgical, neonatal intensive care unit, and pediatric intensive care unit—or their designated representatives. The facility medical director (or designee) will provide information about the facility’s overall characteristics.

#### Sample size.

All four eligible tertiary hospitals in Tanzania will be approached. This approach aligns with global surgery capacity assessments (OReCS, PIPES, WHO SARA), which survey all eligible referral hospitals when the population is small and well‑defined [44].

#### Variables.

Variable domains will be aligned with the Optimal Resources for Children’s Surgery framework across five domains: infrastructure, workforce, service delivery, equipment and supplies, training and governance [28]. Optimal Resources for Children’s Surgery framework‑aligned tool will be used to collect data on the overall facility characteristics and four departments: Paediatric Surgery, neonatal intensive care unit, paediatric intensive care unit, and Anaesthesiology.

Facility‑level variables will include managing authority, level of care, bed capacity, training status, and availability of core services (laboratory, radiology, blood bank, physiotherapy, dietetics). Workforce variables will capture available and required staff across cadres. Service‑delivery variables will include annual and emergency admissions, theatre availability and functionality, and access to paediatric subspecialty consultations. Equipment and supplies will be assessed through availability and functional status of essential items in critical‑care units and paediatric surgical theatres. Governance variables include frequency of service‑delivery meetings and other routine quality‑assurance activities.

The primary outcome will be defined as facility compliance with Optimal Resources for Children’s Surgery standards, which will be summarised using domain‑specific scores, an overall composite score, and quantified resource gaps (absolute shortfalls, percentage deficits, and ordinal deficit scores).

#### Data collection tool and procedures.

The data collection tool was adapted from the Optimal Resources for Children’s Surgery checklist [28] and refined through expert consultation (KL, SM, MKN) to ensure contextual relevance while maintaining fidelity to the domains.

Data will be collected through structured interviews with survey respondents, with the researcher completing the tool based on the information provided and supplementing it with direct observation and verification against inventory records to ensure accuracy and standardisation. See S3 File. Phase III OReCS-Aligned Facility Survey Tool

#### Analysis.

Descriptive statistics using frequencies, percentages, and medians will be used. Resource gaps will be quantified through absolute and relative shortfalls.

Friedman test will assess differences in domain compliance scores across facilities; when significant (p < 0.05), Wilcoxon signed‑rank tests will be utilised for pairwise comparisons between facilities, with Bonferroni‑adjusted p‑values to control for multiple testing. Effect sizes for Wilcoxon tests will be reported using r (40). All computational will be performed in IBM SPSS Statistics version 25 (IBM Corp., Armonk, NY, USA). Reporting of this phase will adhere to Checklist for Reporting Of Survey Studies guidelines [45].

### Phase IV

#### Study design.

Phase IV will be a feasibility assessment evaluating practicality and acceptability of an ARM preoperative checklist.

#### Study population, inclusion and exclusion criteria.

The study population will include medical doctors involved in the perioperative care of children with ARM, specifically Master of Medicine in General Surgery trainees, Paediatric Surgery trainees, Medical Officers, and Paediatric Surgical Specialists.

#### Sample size estimation.

A census approach with consecutive sampling will be used. Based on the staffing structure of the Paediatric Surgical Unit at MNH at the time of study conception, 60 clinicians met the eligibility criteria.

A 60–70% response rate is targeted, which is considered acceptable in this medical practitioners’ participant group [46,47] to provide sufficient information on practicality and acceptability.

#### Variables.

Demographic characteristics (sex, level of training, years in clinical practice) and clinicians’ perceived competence in early ARM assessment, including their ability to evaluate stability, identify associated malformations, and order appropriate investigations, will be captured.

Practicality‑related variables assess how consistently recommended investigations (echocardiography, renal ultrasound, babygram/abdominal pelvic imaging, full blood picture, and serum electrolytes) are completed within 48 hours, as well as clinicians’ perceptions of the accessibility of required subspecialty services.

Acceptability‑related variables evaluate clinicians’ attitudes toward the proposed checklist, including perceived usefulness, documentation burden, training needs, resource requirements, and anticipated impact on time to first surgical intervention. Willingness to adopt and trial the checklist is also measured.

#### Data collection tool and procedures.

Data collection will be conducted through a researcher-administered questionnaire tool composed of Likert‑type items. A context‑specific instrument was developed by the research team (MKN, KL, and SM) and adapted from the validated Acceptability of Intervention Measure and Feasibility of Intervention Measure frameworks [48]. All items were tailored to reflect the workflow for neonates with ARM at MNH. The questionnaire was piloted among four medical doctors, after which minor refinements were made to improve clarity and framing. S4. Phase IV Preoperative Checklist Feasibility Questionnaire (S4 File)

#### Analysis.

Analysis will quantify the practicality and acceptability of the ARM preoperative checklist using data from the Likert‑type questionnaire. Practicality items will be grouped into three indices—burden, utility, and barriers—calculated as mean scores (or medians with interquartile ranges if non‑normal). Internal consistency will be assessed using Cronbach’s alpha, with ≥0.70 indicating acceptable reliability. Group differences across cadre, sex, and years of experience will be examined using one‑way ANOVA when assumptions are met or Kruskal–Wallis tests otherwise. Associations between years of experience and practicality scores will be evaluated using Spearman’s rank correlation, with correlation coefficients and 95% confidence intervals reported.

Acceptability will be assessed through items measuring willingness to use the checklist and perceived impact on time to first surgical intervention. The overall acceptability rate will be defined as the proportion of clinicians expressing strong willingness to adopt the checklist and those who believe it will reduce or not increase time to surgery. Associations between acceptability and cadre, sex, and practicality indices will be analyzed using Chi‑square or Fisher’s exact tests, as appropriate.

Practicality and acceptability scores will be combined into an overall feasibility score, weighted equally (50% each [49]). This equal weighting strategy is methodologically grounded in the principle of equipoisal importance [50], reflecting the theoretical framework that clinical acceptability and operational practicality are equally vital, non-compensatory pre-requisites for intervention sustainability [50]. Feasibility will be categorized as feasible (≥70%), conditionally feasible (50–69%), or not feasible (<50%). All computational procedures performed in IBM SPSS Statistics version 25 (IBM Corp., Armonk, NY, USA). The reporting of this phase will adhere to the Standards for Reporting Implementation Studies guidelines [51].

### Ethical considerations

This study received ethical approval from the University of Cape Town Human Research Ethics Committee (Ref: 262/2024), the Muhimbili National Hospital Institutional Review Board (Ref: MNH/HCRTCU//2024/64), and the National Institute for Medical Research—NaTHREC (Ref: NIMR/HQ/R.8a/Vol.IX/4834).

Written informed consent will be obtained. In Phases I and II, consent will be provided by parents of neonates with ARM, with additional permission for audio‑recording in qualitative interviews. In Phases III and IV, consent will be obtained from facility respondents and pediatric surgical practitioners, respectively. To promote autonomy and minimize coercion, participants may invite an independent witness to observe and attest to the consent process. This option will be offered uniformly, regardless of literacy level, and witnesses will confirm that the study information has been clearly explained and that participation is voluntary.

No personal identifiers will be collected. All data will be stored in secure, password-protected REDCap databases (Vanderbilt University) accessible only to the research team. Audio recordings will be de-identified and kept on encrypted drives, to ensure confidentiality.

### Safety considerations

No clinical interventions will be performed, and procedures will be limited to interviews and questionnaires. There is a risk of emotional distress during qualitative interviews, particularly when discussing pregnancy experiences, care-seeking challenges, or the diagnosis of ARM. To mitigate this, sensitive interviewing techniques will be used, and participants may pause, skip questions, or withdraw at any time. If distress is observed, the Severity of Acute Stress Symptoms—Adult (NSESSS) [52] tool will be used to screen for acute stress symptoms, with referral to appropriate support services when indicated.

### Limitations and mitigation

There is a risk of recall bias for phase I. However, as most of the required information relates to the index pregnancy, the degree of recall bias is anticipated to be limited. Furthermore, the anticipated small sample size may limit precision, reduce power to detect modest associations, and restrict the complexity of multivariable analyses. In phase II, the experiences of parents who did not attend MNH or access healthcare will not be represented. It is likely that their experiences offer unique perspectives on the phenomenon under investigation. Future studies aimed at capturing these unique experiences are recommended.

### Status and timeline of the study

Data collection for all phases began in July 2025. Phase I data collection is expected to conclude in December 2026, while Phases II–IV will conclude in August 2026. Data cleaning, analysis, and the manuscript are expected by February 2027. No results have been generated or analysed at the time of submission of this manuscript.

### Conclusion

The results of this multi-phase mixed-methods study are anticipated to deliver a comprehensive, health-system-wide appraisal of Tanzania’s capacity for early detection and management of major structural birth defects. Several strengths are noteworthy. The study’s conceptual framework provides a structured lens for interpreting results; the use of a relevant tracer condition for major structural birth defects strengthens applicability; and the validity of the methods, including adapted, context‑specific data‑collection tools across phases, enhances reliability. Applying international benchmarks to assess tertiary‑level capacity further supports the generation of robust, trustworthy evidence. Collectively, the results will address the longstanding absence of data that has constrained advocacy, policy development, and service improvement.

Some limitations are anticipated. Anticipated small sample size in Phase I, attributable to the low incidence of ARM, may limit statistical precision. At this stage this limitation has been mitigated by application of non-parametric statistics and limiting the predictors in the multivariable logistic regression analysis, with planned diagnostics reporting to strengthen interpretation of the results. The generalisability of the results will be limited to settings with similar health-system structures.

Findings will be disseminated through policy briefs, stakeholder meetings, scientific conferences, and peer-reviewed publications, with summaries shared with participating facilities to support quality-improvement efforts. Any protocol amendments will be submitted to all approving ethics committees, documented, and communicated to participating sites. Overall, this study will generate actionable evidence to guide resource allocation, strengthen referral and surgical systems, and support targeted interventions aimed at reducing morbidity and mortality among children with major structural birth defects in Tanzania.

## Supporting information

S1 File Phase I Data-collection tool. (PDF)

S2 File Phase II Interview guide. (PDF)

S3 File Phase III OReCS-aligned facility survey tool. (PDF)

S4 File Phase IV Preoperative checklist feasibility questionnaire. (PDF)

S5 File Study protocol GRAMMS checklist. (DOCX)
