# Supplementary material for: An assessment of the health system’s capacity for major structural birth defects care in Tanzania using anorectal malformations as a tracer condition: A multi-phase, mixed-methods study protocol
Source: PLoS One. 2026 Oct 5;21(10):e0359639. doi: 10.1371/journal.pone.0359639 (PMC13637857; doi:10.1371/journal.pone.0359639)
Supplement: Supplementary file 1 [file pone.0359639.s001.pdf]

The evaluation of the current capacity for prevention, and early detection of Birth Defects in Tanzania- Questionnaire for parents and care takers

Record ID ______________________

1. Participant Background Information
1.1 Mothers Information

Age of Participant (years) ______________________
(Age of the participant)

Una umri gani

What was your weight before the current pregnancy (in Kilograms) ______________________
(Weight of the mother before current pregnancy)

Kabla ya ujauzito, ulikua na uzito gani

What is your height (in centi meters) ______________________

Una urefu gani
(Height of the mother)

Did you attend school?
- ◯ Yes
- ◯ No

(Whether the mother attended school)

Je, ulihudhuria shule?

If you attended school, what is the highest level of school you attended?

Ni kiwango gani cha juu cha shule ulichohudhuria?
- ◯ Pre-primary
- ◯ Primary
- ◯ Post-primary
- ◯ Secondary "O" level
- ◯ Post-secondary "O" level training
- ◯ Secondary "A" level training
- ◯ Post-secondary "A" level training
- ◯ University
- ◯ I don't Know

(What is the highest level of school you attended)

1. Participant Background Information
1.2 Marital status

Are you currently married or living together with a man as if married?
- ◯ Yes currently Married
- ◯ Yes living with a man
- ◯ No not in union

(Current marital status at time of interview)

Je, wewe kwa sasa, upo kwenye ndoa au unaishi pamoja na mzazi mwenzako?

Have you ever been married or lived together with a man as if married?<br><br>Umewahi kuishi katika ndoa au kuishi pamoja na mzazi mwenzako?	◯ Yes formerly married<br>◯ Yes lived with a man<br>◯ No
What is your marital status now?<br><br>Kwa sasa, kimahusiano, wewe ni: mjane, mtalaka, au mmetengana?	◯ Widowed<br>◯ Divorced<br>◯ Separated<br>(what is your marital status now)

2. Home and Environmental Exposures

What is the cooking fuel used at home?<br><br>Aina gani ya mafuta inatumika kupikia nyumbani?	◯ Solid cooking fuel<br>◯ Clean cooking fuel<br>(Solid fuels- coal/lignite, charcoal, wood, straw/shrub/grass, agricultural crops, and animal dung. Clean fuels- electricity, liquefied petroleum gas (LPG), natural gas, and biogas.)
Do you currently smoke cigarettes every day, some days, or not at all?<br><br>Je, kwa sasa unavuta sigara kila siku, siku fulani, au la?	◯ Every day<br>◯ Some Days<br>◯ Not at all<br>(Do you smoke)
If you smoke, on average, how many cigarettes do you currently smoke each day?<br><br>Kama unavuta sigara, kwa wastani, ni sigara ngapi unavuta kila siku?	__________________________________<br>(How many cigarettes do you smoke)
Have you ever consumed a drink that contains alcohol such as beer, wine, spirit, fermented cider or local brewers such as mbege, ulanzi, gongo/chang'aa etc?<br><br>Je, umewahi kunywa kinywaji chenye pombe kama bia, divai, pombe kali, au pombe za kienyeji kama vile mbege, ulanzi, gongo/chang'aa n.k?	◯ Yes<br>◯ No<br>(Ever Drank alcohol)
In the past 12 months, how frequently have you had at least one alcoholic drink?<br><br>Katika miezi 12 iliyopita, kwa wastani ni mara ngapi umekunywa kinywaji hichi?	◯ Daily<br>◯ Weekly<br>◯ Monthly<br>◯ Less than monthly<br>◯ Never drunk<br>(Last 12 months, how frequently one took alcohol)
Do you have any chronic medical conditions?<br><br>Je, una magonjwa yoyote ya muda mrefu?	◯ Yes<br>◯ No<br>(Any chronic medical conditions)

Which chronic medical conditions do you have?<br><br>Una magonjwa gani?	☐ Hypertension<br>☐ Diabetes<br>☐ HIV<br>☐ TB/ Chronic cough<br>☐ Arthritis/ Joint aches<br>☐ Epilepsy<br>☐ Other medical illness/Condition<br>(which comorbid)
Name other medical condition not listed above<br><br>Taja ugonjwa huu	____________________<br>(Name other comorbid)
Have you been using regular medications within 12 months prior to, or during the most recent pregnancy?<br><br>Je, umekuwa ukitumia dawa ndani ya miezi 12 kabla ya, au wakati wa ujauzito wa hivi karibuni?	◯ Yes<br>◯ No<br>(Medications include- Herbal remedies, those given by traditional healer, and health facilities. Dawa za miti shamba, zilizo tolewa na mganga wa kienyeji, au kutoka vituo vya afya)
In the past 12 months prior to, or during the most recent pregnancy, how frequently were you using these medications?<br><br>Katika miezi 12 iliyopita kabla ya, au wakati wa ujauzito wa hivi karibuni, ni mara ngapi ulikuwa unatumia dawa hizi?	◯ Daily<br>◯ Weekly<br>◯ Monthly<br>◯ Less than monthly<br>(how frequently have you been using the medications)
Where were these medications obtained from?<br><br>Dawa hizi zilipatikana wapi?	☐ Traditional healer premises<br>☐ Over the counter, drug store<br>☐ Health facility/ Hospital<br>(where did you obtain the medications)

Does your employment/ economic activity place you in contact with the following?

Je, ajira/shughuli zako za kiuchumi zinakuweka katika ukaribu na yafuatayo?

	Yes	No	I dont know
Industrial chemicals	☐	☐	☐
Textile chemicals	☐	☐	☐
Fertilizer chemicals/ Pesticides	☐	☐	☐
Heavy metals/Mining	☐	☐	☐

3. Preconception Care

Have you ever lost a pregnancy?<br><br>Umewahi kupoteza ujauzito?	◯ Yes<br>◯ No<br>(Ever lost a pregnancy)
How many times have you lost a pregnancy?<br><br>Umepoteza ujauzito mara ngapi?	____________________<br>(how many pregnanices lost)

How many times have you given live births?

Ni mara ngapi umezaa watoto hai?

____
(Number of live births)

What was your age at first pregnancy? (Years)

Ulikua una umri gani wakati wa ujauzito wako wa kwanza?

____
(Age at first pregancy)

What was your age at first live birth? (Years)

Ulikua una umri gani wakati wa kujifungua moto hai kwa mara ya kwanza?

____
(Age at first live birth)

What was your age at the latest birth? (Years)

Ulikua una umri gani wakati unajifungua ujauzito huu wa karibuni?

____
(Age at latest livebirth)

Before the most recent pregnancy, did you visit a health facility for yourself, or did a healthcare worker visit you to discuss your health?

Kabla ya ujauzito wa hivi karibuni, ulitembelea kituo cha afya kwa ajili yako mwenyewe, au mfanyakazi wa afya alikutembelea kujadili afya yako?

◯ Yes
◯ No
(Did you have a preconception visit)

Please indicate whether the following were discussed during the visit
(Tafadhali onyesha kama yafuatayo yalijadiliwa wakati wa ziara hiyo)

	Yes	No	I don't know
Family planning and contraception (Uzazi wa mpango)	☐	☐	☐
Nutrition and weight (Lishe na uzito)	☐	☐	☐
Alcohol and smoking cessation (Kuacha wa pombe na sigara)	☐	☐	☐
I was asked if I have chronic medical diseases (Niliulizwa kama nina magonjwa sugu ya kiafya)	☐	☐	☐
Environmental risks to pregnancy (Hatari za mazingira kwa ujauzito)	☐	☐	☐
Inheritable conditions and birth defects (Magonjwa ya kurithi na kasoro za kumaumbile)	☐	☐	☐

Medications and pregnancy risk (Athari za dawa kwa ujauzito)	☐	☐	☐

Please indicate whether the following were provided before this current pregnancy (Tafadhali onyesha kama yafuatayo yalitolewa kabla ya ujauzito huu wa sasa)

	Yes	No	I dont know
Tetanus Toxoid Vaccine (Chanjo ya pepopunda)	☐	☐	☐
Syphilis screening (RPR) (kupima kaswende)	☐	☐	☐
Rubella screening (kupima rubella)	☐	☐	☐

2. Antenatal Care (Review ANC card)

Did you see anyone for antenatal care for this pregnancy?

Je, ulionana na mtu yeyote juu ya afya katika ujauzito huu?

◯ Yes
◯ No
(Attended antenatal care)

Whom did you see? (select the highest type of provider)

Ulimwona nani? (Chagua aina ya juu zaidi ya mtoa huduma)

◯ Doctor/ AMO
◯ Clinical Officer
◯ Assistant Clinical Officer
◯ Nurse/ Midwife
◯ Assistant Nurse
◯ MCH Aide
◯ Community Health Worker
◯ Trained TBA/TBA
◯ I do not know/ remember
(Who did you see at ANC visit)

Where did you receive antenatal care for this pregnancy?
(Apart from facility level, note whether Government/Parastatal, Religious/Voluntary, or Private facility)

Ulipata wapi huduma ya afya ya ujauzito huu?
(kituo cha afya cha uma, dini/kujitolea, au binasi)

- ◯ National/Zonal/Referral/ Specialized Hospital-Government or Parastatal
- ◯ Regional Referral Hospital-Government or Parastatal
- ◯ Regional Hospital-Government or Parastatal
- ◯ District Hospital-Government or Parastatal
- ◯ Health Centre-Government or Parastatal
- ◯ Dispensary-Government or Parastatal
- ◯ Clinic-Government or Parastatal
- ◯ CHW-Government or Parastatal
- ◯ Referral/ Specialized Hospital
- ◯ District Hospital-Religious/ Voluntary
- ◯ Hospital-Religious/ Voluntary
- ◯ Health Centre-Religious/ Voluntary
- ◯ Dispensary-Religious/ Voluntary
- ◯ Clinic-Religious/ Voluntary
- ◯ Specialized Hospital-Private
- ◯ Hospital-Private
- ◯ Health Centre-Private
- ◯ Dispensary-Private
- ◯ Clinic-Private
- ◯ I don't know
- ◯ Other facility, unsure of facility

(note the type of facility)

If unsure of facility type, mention facility name

____
(other facility type for ANC)

How many months pregnant were you when you first received antenatal care for this pregnancy? (Months pregnant)

Ulikuwa na ujauzito wa miezi mingapi wakati ulipopata huduma ya afya ya ujauzito kwa mara ya kwanza?

____

How many times did you receive antenatal care during this pregnancy?

Ni mara ngapi ulihduria kliniki ya huduma ya fya ya ujauzito katika ujauzito wa mtoto huyu?

____
(number of ANC visits)

As part of your antenatal care during this pregnancy, were any of the following done at least once:

(Kama sehemu ya huduma ya afya ya ujauzito, katika ujauzito huu, ya yafuatayo yalifanywa angalau mara moja:)

	Yes	No	I don't know
Was your blood pressure measured?	☐	☐	☐
Did you give a urine sample?	☐	☐	☐

Did you give a blood sample?	☐	☐	☐

During this pregnancy, was the following done/offered?
(Wakati wa ujauzito huu, je, yafuatayo yalitolewa/ kufanywa)

	Yes	No	I don't know
Antenatal (obstretic) ultrasound	☐	☐	☐
Antenatal (Fetal) scan ultrasound	☐	☐	☐
Amniocentesis for screening	☐	☐	☐
Chorionic villous sampling	☐	☐	☐
Iron supplementation	☐	☐	☐
Folic Acid supplementation	☐	☐	☐

3. Delivery information
(Review in ANC card where available)

Where did you give birth to your baby?

Ulijifungua wapi ujauzito huu wa karibuni

- ◯ Her Home
- ◯ Other Home
- ◯ TBA Premises
- ◯ National/ Zonal/ Specialized Hospital-Government/ Parastatal
- ◯ Regional Referral Hospital-Government/ Parastatal
- ◯ Regional Hospital-Government/ Parastatal
- ◯ District Hospital-Government/ Parastatal
- ◯ Health Centre-Government/ Parastatal
- ◯ Dispensary-Government/ Parastatal
- ◯ Referral/Specialized Hospital- Religious/ Voluntary
- ◯ District Hospital- Religious/ Voluntary
- ◯ Hospital- Religious/ Voluntary
- ◯ Health Centre- Religious/ Voluntary
- ◯ Dispensary- Religious/ Voluntary
- ◯ Clinic- Religious/ Voluntary
- ◯ Specialized Hospital- Private
- ◯ Hospital- Private
- ◯ Health Centre- Private
- ◯ Dispensary- Private
- ◯ Clinic- Private
- ◯ Other facility, unsure of facility

If unsure of facilty type, mention name

Kama hauna uhakika, tafadhali taja kituo

____
(other facility name)

Who assisted with the delivery of your baby?

Ni nani aliyesaidia katika kujifungua mtoto wako?

- ◯ Doctor/ AMO- Health Personnel
- ◯ Clinical Officer- Health Personnel
- ◯ Assistant Clinical Officer- Health Personnel
- ◯ Nurse/ Midwife- Health Personnel
- ◯ Community Health Worker- Other Person
- ◯ Trained TBA/TBA- Other Person
- ◯ Relative/ Friend- Other Person
- ◯ I do not know/ remember
- ◯ Other

If other, please mention<br><br>Kama nyingine, tafadhali taja	__________________________________
What was the mode of delivery?<br><br>Ulijifungua kwa njia gani?	◯ Normal vaginal delivery<br>◯ Vacuum/ forceps assisted delivery.<br>◯ Caesarean section<br>◯ I do not know/ remember<br>(mode of delivery)

4. Post natal care
(Review card if available)

How long after your baby was delivered did you stay at the place of birth?<br><br>Baada ya kujifungua, ulikaa kwa muda gani sehemu uliyojifungulia kabla ya kurudi nyumbani kwako?	◯ Less than 24 hours<br>◯ 1 day<br>◯ 2 days<br>◯ More than 2 days<br>(length of stay after delivery)
If more than 2 days, state the reason<br><br>Kama zaidi ya siku mbili, taja sababu	◯ Maternal medical reason<br>◯ Newborn medical reason<br>◯ Other reason
If other reason for more than 2 days after delivery, state reason<br><br>Kama kuna sababu nyingine ya kulazwa zaidi ya siku mbili baada ya kujifungua, tafadhali itaje	__________________________________
Did anyone check on your health while you were still in the facility?<br><br>Je, kuna mtu yeyote aliyechunguza afya yako kabla ya kurhusiwa kuondoka sehemu uliyo jifungulia na kurudi nyumbani?	◯ Yes<br>◯ No
How long after delivery did your first check take place? (in hours)<br><br>Ni muda gani baada ya kujifungua, ulipimwa afya yako kwa mara ya kwanza?	__________________________________<br>(Ni muda gani baada ya kujifungua, ulipimwa afya yako kwa mara ya kwanza?)

Which health personnel checked on your health at that time?<br><br>Ni wahudumu gani wa afya waliochunguza afya yako wakati huo?	◯ Doctor/ AMO- Health Personnel<br>◯ Clinical Officer- Health Personnel<br>◯ Assistant Clinical Officer- Health Personnel<br>◯ Nurse/ Midwife- Health Personnel<br>◯ Assistant Nurse- Health Personnel<br>◯ MCH Aide- Health Personnel<br>◯ Community Health Worker- Other Person<br>◯ Trained TBA/TBA- Other Person<br>◯ Relative/ Friend- Other Person<br>◯ I don't know/remember<br>(Ni wahudumu gani wa afya waliochunguza afya yako wakati huo?)
Did anyone check on your baby's health while you were still in the facility<br><br>Je, kuna mtu yeyote aliyechunguza afya ya mtoto wako kabla ya kurhusiwa kuondoka sehemu uliyo jifungulia na kurudi nyumbani?	◯ Yes<br>◯ No
How long after delivery was your baby's health first checked? (In hours)<br><br>Ni muda gani baada ya kujifungua, afya yam toto wako alichunguzwa kwa mara ya kwanza?	______________________
Which health personnel checked on your baby's health at that time?<br><br>Ni muhudumu gani wa afya waliochunguza afya ya mtoto wako wakati huo?	◯ Doctor/ AMO- Health Personnel<br>◯ Clinical Officer- Health Personnel<br>◯ Assistant Clinical Officer- Health Personnel<br>◯ Nurse/ Midwife- Health Personnel<br>◯ Assistant Nurse- Health Personnel<br>◯ MCH Aide- Health Personnel<br>◯ Community Health Worker- Other Person<br>◯ Trained TBA/TBA- Other Person<br>◯ Relative/ Friend- Other Person<br>◯ I don't know/remember
Where did this check of your baby take place?<br><br>Uchunguzi huu wa mtoto wako ulifanyika wapi?	◯ Her Home<br>◯ Other Home<br>◯ TBA Premises<br>◯ National/ Zonal/ Specialized Hospital-Government/ Parastatal<br>◯ Regional Referral Hospital-Government/ Parastatal<br>◯ Regional Hospital-Government/ Parastatal<br>◯ District Hospital-Government/ Parastatal<br>◯ Health Centre-Government/ Parastatal<br>◯ Dispensary-Government/ Parastatal<br>◯ Referral/Specialized Hospital- Religious/ Voluntary<br>◯ District Hospital- Religious/ Voluntary<br>◯ Hospital- Religious/ Voluntary<br>◯ Health Centre- Religious/ Voluntary<br>◯ Dispensary- Religious/ Voluntary<br>◯ Clinic- Religious/ Voluntary<br>◯ Specialized Hospital- Private<br>◯ Hospital- Private<br>◯ Health Centre- Private<br>◯ Dispensary- Private<br>◯ Clinic- Private<br>◯ Other facility, unsure of facility
If unsure of facility type, provide name please	______________________

During the first two days after your baby's birth, did any healthcare provider do the following:

Katika siku mbili za kwanza baada ya kuzaliwa kwa mtoto wako, kuma mtoa huduma yeyote wa afya alifanya yafuatayo:

	Yes	No	I don't know
Examine the cord	○	○	○
Measure your baby's temperature	○	○	○
Counsel you on danger signs for newborns	○	○	○
Counsel you on breastfeeding	○	○	○
Observe breastfeeding	○	○	○

I am going to read the following statements, tell me how much you agree with me. During the baby check the following were done

Nitasoma taarifa zifuatazo, niambie ni kiasi gani unakubaliana nami. Wakati wa ukaguzi wa mtoto, yafuatayo yalifanywa

	Strongly Agree	Agree	Neutral	Disagree	Strongly Disagree
The general appearance was examined	○	○	○	○	○
The head circumference was measured	○	○	○	○	○
The chest area was auscultated	○	○	○	○	○
The abdomen was examined	○	○	○	○	○
The genitalia were examined	○	○	○	○	○
The anal area was examined	○	○	○	○	○
The limbs were examined	○	○	○	○	○
Told baby is fine	○	○	○	○	○

How long after birth was it noticed that your baby can not pass stools? (in Hours) ______________________________

Ni muda gani baada ya kuzaliwa iligundulika kuwa mtoto wako hawezi kupitisha haja kubwa?

Which person noticed that your baby does not have a normal anal opening?<br><br>Ni nani aliyegundua kuwa mtoto wako hana njia ya kawaida ya haja kubwa?	◯ Doctor/ AMO- Health Personnel<br>◯ Clinical Officer- Health Personnel<br>◯ Assistant Clinical Officer- Health Personnel<br>◯ Nurse/ Midwife- Health Personnel<br>◯ Assistant Nurse- Health Personnel<br>◯ MCH Aide- Health Personnel<br>◯ Community Health Worker- Other Person<br>◯ Trained TBA/TBA- Other Person<br>◯ Relative/ Friend- Other Person<br>◯ Myself<br>◯ I don't know/remember
Where was it detected that your baby does not have a normal anal opening?<br><br>Ni wapi iligundulika kuwa mtoto wako hana njia ya kawaida ya haja kubwa?	◯ Her Home<br>◯ Other Home<br>◯ TBA Premises<br>◯ National/ Zonal/ Specialized Hospital-Government/ Parastatal<br>◯ Regional Referral Hospital-Government/ Parastatal<br>◯ Regional Hospital-Government/ Parastatal<br>◯ District Hospital-Government/ Parastatal<br>◯ Health Centre-Government/ Parastatal<br>◯ Dispensary-Government/ Parastatal<br>◯ Referral/Specialized Hospital- Religious/ Voluntary<br>◯ District Hospital- Religious/ Voluntary<br>◯ Hospital- Religious/ Voluntary<br>◯ Health Centre- Religious/ Voluntary<br>◯ Dispensary- Religious/ Voluntary<br>◯ Clinic- Religious/ Voluntary<br>◯ Specialized Hospital- Private<br>◯ Hospital- Private<br>◯ Health Centre- Private<br>◯ Dispensary- Private<br>◯ Clinic- Private<br>◯ Other facility, unsure of facility
If unsure of facility type, kindly mention facility name<br><br>Kama hauna uhakika, tafadhali taja jina la kituo/mahali	__________________________________
How long after birth did it take to reach the referral hospital for your baby's health? (number in days)<br><br>Ilichukua muda gani kufika hospitali ya rufaa kwa ajili ya matibabu ya mtoto wako?	__________________________________
