# Supplementary material for: An assessment of the health system’s capacity for major structural birth defects care in Tanzania using anorectal malformations as a tracer condition: A multi-phase, mixed-methods study protocol
Source: PLoS One. 2026 Oct 5;21(10):e0359639. doi: 10.1371/journal.pone.0359639 (PMC13637857; doi:10.1371/journal.pone.0359639)
Supplement: Supplementary file 2 [file pone.0359639.s002.pdf]

Interview Guide

Major Structural Birth Defects in Tanzania: An Assessment of the Health System's Capacity Using Anorectal Malformations

Access to Care Experiences of Parents with neonates with anorectal malformations in Tanzania

Time of Starting Interview:

Time of Finishing Interview:

Date: Place:

Interviewer:

Interviewee Identification Number/Alias:

INTRODUCTION

Thank you for consenting to participate in this study.

QUESTIONS

1. Can you describe how you found out your child has anorectal malformation?
2. Can you describe how you felt?
3. Can you describe how your partner reacted?
4. Can you describe how your relatives reacted?
5. Can you describe what decisions you considered and made about your child's condition?
6. Can you mention what treatment options you considered?
    a. Probe for traditional healers roles
    b. Probe for faith healers' roles
    c. Probe for advice from relatives
    d. Probe for the description of the decision to visit the hospital
7. Can you describe how the healthcare workers reacted?
8. What did you think of the treatment suggested at the hospital
    a. Probe for the care giver's impression of prognosis
    b. Probe for cost implications and preparedness, whether they have health insurance
9. Can you describe the cost of treatment proposed by the hospital
10. How has seeking healthcare affected your ability to earn a living
    a. Probe for mitigations
11. How has seeking health care for this child affected your other children?
12. How has seeking health care for this child affected your partner/husband?
    a. Probes for real examples, roles played in examples, associated feelings and outcomes.
    b. Allow uninterrupted explanations

13. What is the status of your child now
    a. *Probe for continence, constipation*
    b. *Probe for methods to address any of the above issues that the care giver used*
14. How has this affected her/his ability to attend school
    a. *Probe for financial constraints, health concerns, stigma*
15. How has this affected his/her ability to interact with other children
    a. *Probe for stigma, isolation, depressed mood, development*
16. Since the birth of your child until now, how do you describe the health care you received
    a. *Probe for positive and negative aspects of care before reaching the referral hospital*
    b. *Probe for positive and negative aspects of surgical care received*
    c. *Probe for positive and negative aspects of follow up*

Field Notes:

Thank the participants for their participation in this study. Assure the participant that all responses will be confidential and that personal identification is protected.
