# Supplementary material for: An assessment of the health system’s capacity for major structural birth defects care in Tanzania using anorectal malformations as a tracer condition: A multi-phase, mixed-methods study protocol
Source: PLoS One. 2026 Oct 5;21(10):e0359639. doi: 10.1371/journal.pone.0359639 (PMC13637857; doi:10.1371/journal.pone.0359639)
Supplement: Supplementary file 3 [file pone.0359639.s003.pdf]

The Evaluation Of The Tertiary Level Care Capacity

Record ID ________________________________

1. Facility Characteristics

The type of respondent to the general facility questions
- ◯ Medical Director
- ◯ Head of Clinical Department
- ◯ Nurse Manager
- ◯ Other

If other, please specify ________________________________

Managing Authority of the facility
- ◯ Faith-Based Organization/ Parastatal
- ◯ Voluntary Agency
- ◯ Private Sector
- ◯ Public Sector
- ◯ Other/ Unsure

If unsure about facility, please mention the facility name for cross checking ________________________________

Level of Facility
- ◯ National Referral Hospital
- ◯ Zonal Referral Hospital
- ◯ Regional referral hospital
- ◯ District-level Hospital
- ◯ Other/ Unsure

Total facility Bed capacity ________________________________

Pediatrics bed capacity ________________________________
(General peads- in some facilities/)

Pediatric surgery bed capacity ________________________________

Facility PICU Bed capacity ________________________________

Facility NICU Bed capacity ________________________________

Location of facility- By region	◯ Arusha Region<br>◯ Dar es Salaam Region<br>◯ Dodoma Region<br>◯ Geita Region<br>◯ Iringa Region<br>◯ Kagera Region<br>◯ Katavi Region<br>◯ Kigoma Region<br>◯ Kilimanjaro Region<br>◯ Lindi Region<br>◯ Manyara Region<br>◯ Mara Region<br>◯ Mbeya Region<br>◯ Morogoro Region<br>◯ Mtwara Region<br>◯ Mwanza Region<br>◯ Njombe Region<br>◯ Pemba North Region<br>◯ Pemba South Region<br>◯ Pwani Region<br>◯ Rukwa Region<br>◯ Ruvuma Region<br>◯ Shinyanga Region<br>◯ Simiyu Region<br>◯ Singida Region<br>◯ Songwe Region<br>◯ Tabora Region<br>◯ Tanga Region<br>◯ Unguja North Region<br>◯ Unguja South Region<br>◯ Zanzibar Central/South Region
Is this a facility used for the training of medical professionals	◯ Yes<br>◯ No
If so, which health professional cadres train at this site- tick all that apply	☐ Health professionals Certificate level training<br>☐ Health professionals Diploma level training<br>☐ Health professionals Degree level training<br>☐ Health professionals Masters level training<br>☐ Clinical Specialties Masters level training

Does this facility offer the following services? If a child requires these services, can they be offered in this facility?

	Yes	No
Paediatric surgery	◯	◯
Paediatric anaesthesiology	◯	◯
Neonatal intensive care	◯	◯
Pediatrics intensive care	◯	◯
Paediatrics radiology services	◯	◯
Paediatric urology services	◯	◯
Paediatric cardiology services	◯	◯
Chemistry laboratory	◯	◯

Haematology laboratory	◯	◯
Blood bank service	◯	◯
Microbiology laboratory	◯	◯
Physio/rehabilitative service	◯	◯
Dietetics service	◯	◯

PICU Data on

Staffing, Service and Quality, Research and Training and Work load

Respondent of PICU Service questions
- ◯ Medical Director
- ◯ Head of Clinical Department
- ◯ Nurse Manager
- ◯ Other

In the PICU, how many Pediatric Intensivist- are available ________________________________

Currently, how many pediatric intensivists have been requested by the PICU unit ________________________________

In the PICU, how many General pediatricians- are available ________________________________

Currently, how many general pediatricians have been requested by the PICU unit ________________________________

In the PICU, how many General Medical Doctors/registrars (MD, MMED Trainees)- are available ________________________________

Currently, how many registrars/MD have been requested by the PICU unit ________________________________

In the PICU, how many assistant medical officers are available ________________________________

Currently, how many assistant medical officers have been requested by the PICU unit ________________________________

In the PICU, how many Registered nurses- are available ________________________________

Currently, how many registered nurses have been requested by the PICU unit ________________________________

In the PICU, how many Enrolled nurses- are availlable ________________________________

Currently, how many enrolled nurses have been requested by the PICU unit ________________________________

In the PICU, how many nurse assistants/Attendants- are available ________________________________

Currently, how many nurse assistants/attendants have been requested by the PICU unit	______________
In the last year, what was the total number of PICU admissions?	______________
In the last year, what was the total number of emergency PICU admissions?	______________

In the last 6 months, how regularly do the following meetings occur in PICU

	Always	Sometimes	Every once in a while	Rarely	Never
Department/Unit Meetings for Service Delivery	◯	◯	◯	◯	◯
Morbidity and Mortality Meetings	◯	◯	◯	◯	◯
Grand Round/Clinical meetings	◯	◯	◯	◯	◯
CME/CPD activities	◯	◯	◯	◯	◯

In the last 6 months, how regularly do the following occur in PICU

	Always	Sometimes	Every once in a while	Rarely	Never
Regular clinical reports	◯	◯	◯	◯	◯
Audit reports	◯	◯	◯	◯	◯
Quality improvement projects	◯	◯	◯	◯	◯
Other research	◯	◯	◯	◯	◯
Maintain an admissions database	◯	◯	◯	◯	◯

Pediatric Surgery Data on

Staffing, Service and Quality, Research and Training and Work Load

Respondent of Pediatric Surgery Questions	◯ Medical Director<br>◯ Head of Clinical Department<br>◯ Nurse Manager<br>◯ Other
In the Pediatric Surgery Service, how many Pediatric Surgeon- are available	______________
In the Pediatric Surgery service, how many Pediatric Surgeons- have been requested by the unit	______________
In the Pediatric Surgery service, how many General Surgeons- are available	______________
In the Pediatric Surgery service, how many Pediatric Surgeon- have been requested by the unit	______________
In the Pediatric Surgery service, how many Medical doctors/Registrars- are available	______________

In the Pediatric Surgery service, how many Medical doctors/Registrars- have been requested by the unit	__________________
In the Pediatric Surgery service, how many assistant medical officers- are available	__________________
In the Pediatric Surgery service, how many assistant medical officers have been requested by the unit	__________________
In the Pediatric Surgery Service, how many registered nurses- are available	__________________
In the Pediatric Surgery service, how many registered nurses have been requested by the unit	__________________
In the Pediatric Surgery Service, how many enrolled nurses- are available	__________________
In the Pediatric Surgery Service, how many enrolled nurses- have been requested by the unit	__________________
In the Pediatric Surgery Service, how many nurse assistants/ attendants are available	__________________
In the Pediatric Surgery Service, how many nurse assistants/attendants have been requested by the unit	__________________
In the last year, how many pediatric surgical patients were admitted in the unit?	__________________
In the last year, what was the total number of emergency admissions to pediatric surgical unit?	__________________

In the last 6 months, how regularly do the following meetings occur in pediatric surgery Service

	Always	Sometimes	Every once in a while	Rarely	Never
Service Delivery Meetings	○	○	○	○	○
Morbidity and Mortality Meetings	○	○	○	○	○
Grand Round/Clinical Meetings	○	○	○	○	○
CME/CPD Meetings	○	○	○	○	○

In the last 6 months, how regularly do the following occur in Pediatric Surgery

	Always	Sometimes	Every once in a while	Rarely	Never
Regular clinical reports	○	○	○	○	○
Audit reports	○	○	○	○	○

Quality improvement projects	◯	◯	◯	◯	◯
Other research	◯	◯	◯	◯	◯
Maintain an admission database	◯	◯	◯	◯	◯

Anasthesiology Department Data on

Staffing, Service and Quality, Research and Training and Work Load

Respondent to Anesthesiology questions
◯ Medical Director
◯ Head of Clinical Department
◯ Nurse Manager
◯ Other

In the Anesthesia service, how many Pediatric Anesthesiologists are available
____

In the Anesthesia service, how many Pediatric Anesthesiologists have been requested by the unit
____

In the Anesthesia service, how many General Anesthesiologists are available
____
(Specialist Anasthesiologist)

In the Anesthesia service, how many General Anesthesiologists have been requested by the unit
____
(Specialist Anesthesiologist)

In the Anesthesia service, how many Medical doctors/registrars are available
____

In the Anesthesia service, how many medical doctors/ registrars have been requested by the unit
____

In the Anesthesia service, how many nurse-anesthetists are available
____

In the Anesthesia service, how many nurse-anesthetists have been requested by the unit
____

In the Anesthesia service, how many nurse officers are available
____

In the Anesthesia service, how many nurse officers have been requested by the unit
____

In the Anesthesia service, how many nurse attendants are available
____

In the Anesthesia service, how many nurse attendants have been requested by the unit
____

In the last year, what was the total number of pediatric cases operated in that year?
____

In the last 6 months, how regularly do the following meetings occur in Anasthesia service

	Always	Sometimes	Every once in a while	Rarely	Never
Service Delivery Meetings	◯	◯	◯	◯	◯
Morbidity and Mortality Meetings	◯	◯	◯	◯	◯
Grand Round/Clinical Meetings	◯	◯	◯	◯	◯
CME/CPD	◯	◯	◯	◯	◯

In the last 6 months, how regularly do the following occur in anesthesiology department

	Always	Sometimes	Every once in a while	Rarely	Never
regular clinical reports	◯	◯	◯	◯	◯
Audit reports	◯	◯	◯	◯	◯
Quality improvement Projects	◯	◯	◯	◯	◯
Other research	◯	◯	◯	◯	◯
Maintain database of cases	◯	◯	◯	◯	◯

NICU Data on

Staffing, Service and Quality, Research and Training and Work Load

Respondentt to NICU questions

◯ Medical Director
◯ Head of Clinical Department
◯ Nurse Manager
◯ Other

In the NICU service, how many neonatologists are available

____

In the NICU service, how many neonatologists have been requested by the unit

____

In the NICU service, how many pediatricians are available

____

In the NICU service, how many pediatricians have been requested by the unit

____

In the NICU service, how many medical officers/registrars are available

____

In the NICU service, how many medical officers/registrars have been requested

____

In the NICU service, how many registered nurses are available

____

In the NICU service, how many registered nurses have been requested

____

In the NICU service, how many enrolled nurses are available

____

In the NICU service, how many enrolled nurses have been requested by the unit	______________________
In the NICU service, how many nurse assistants/ attendants are available	______________________
In the NICU service, how many nurse assistants/attendants have been requested by the unit	______________________
In the last year, what was the total number of NICU admissions?	______________________

In the last 6 months, how regularly do the following meetings occur in NICU service

	Always	Sometimes	Every once in a while	Rarely	Never
Service Delivery Meetings	◯	◯	◯	◯	◯
Morbidity and Mortality Meetings	◯	◯	◯	◯	◯
Grand Rounds/Clinical meetings	◯	◯	◯	◯	◯
CME/CPD	◯	◯	◯	◯	◯

In the last 6 months, how regularly do the following occur in NICU

	Always	Sometimes	Every once in a while	Rarely	Never
Regular clinical reports	◯	◯	◯	◯	◯
Audit reports	◯	◯	◯	◯	◯
Quality Improvement Project	◯	◯	◯	◯	◯
Other research	◯	◯	◯	◯	◯
Maintain an admission database	◯	◯	◯	◯	◯

NICU- Availability of essential equipment and facilities

In the NICU unit, how many Neonatal Mechanical Ventilators are available	______________________
In the NICU unit, if Neonatal Mechanical ventilators are available, what is the functional status	◯ Fully functional<br>◯ Occasionally functional<br>◯ Not functional
In the NICU unit, how many Neonatal Mechanical Ventilators have been requested by the unit	______________________
In the NICU unit, how many beds have wall monitors for real-time vital monitoring	______________________
In the NICU unit, if there are beds with wall monitors for real-time vital monitoring, how often do the wall monitors work?	◯ Fully functional<br>◯ Occasionally functional<br>◯ Not functional
In the NICU unit, how many beds with wall monitors have been requested by the unit?	______________________

In the NICU unit, how many beds have radiant warmers and thermoregulation capacity?	__________________________
In the NICU unit, if there are beds have radiant warmers and thermoregulation capacity, how often are they functional?	◯ Fully functional<br>◯ Occasionally functional<br>◯ Not functional
In the NICU unit, how many beds have radiant warmers and thermoregulation capacity have been requested by the unit	__________________________
In the NICU unit, how many neonatal-sized laryngoscopes in case invasive ventilation is required?	__________________________
In the NICU unit, if there are there neonatal-sized laryngoscopes, how often are they functioning?	◯ Fully functional<br>◯ Occasionally functional<br>◯ Not functional
In the NICU unit, how many neonatal-sized laryngoscopes have been requested by the unit?	__________________________
In the NICU unit, is there access to a blood gas analyzer?	◯ Yes<br>◯ No
If a blood gas analyzer is accessible in the NICU, how often is it functioning?	◯ Fully functional<br>◯ Occasionally functional<br>◯ Not functional

Availability of NICU infrastructure

	Always	Sometimes	Every once in a while	Rarely	Never
Oxygen availability	◯	◯	◯	◯	◯
Negative pressure suction facility	◯	◯	◯	◯	◯
Neonatal Ambu-bags	◯	◯	◯	◯	◯
Neonatal Endo tracheal tubes	◯	◯	◯	◯	◯
Vassopressor drugs for resuscitation	◯	◯	◯	◯	◯
Pulse oximeter	◯	◯	◯	◯	◯
Thermometer	◯	◯	◯	◯	◯
Weighing Scale	◯	◯	◯	◯	◯
Glucometer	◯	◯	◯	◯	◯
Portable X ray machine	◯	◯	◯	◯	◯
Portable Ultrasound Machine	◯	◯	◯	◯	◯

PICU- Availability of essential equipment and facilities

In the PICU unit, how many Pediatric Mechanical Ventilators are available

____

In the PICU unit, if Pediatric Mechanical ventilators are available, what is the functional status

◯ Fully functional
◯ Occasionally functional
◯ Not functional

In the PICU unit, how many Pediatric Mechanical Ventilators have been requested by the unit?

____

In the PICU unit, how many beds have wall monitors for real-time vital monitoring

____

In the PICU unit, if beds with wall monitors for real-time vital monitoring are available, how often do the monitors function

◯ Fully functional
◯ Occasionally functional
◯ Not functional

In the PICU unit, how many beds have wall monitors for real-time vital monitoring have been requested by the unit

____

In the PICU unit, how many pediatric-sized laryngoscopes in case invasive ventilation is required are available

____

In the PICU unit, if there are pediatric-sized laryngoscopes, how often are they functioning?

◯ Fully functional
◯ Occasionally functional
◯ Not functional

In the PICU unit, how many pediatric-sized laryngoscopes in case invasive ventilation is required have been requested by the unit

____

In the PICU, is there access to a blood gas analyzer?

◯ Yes
◯ No

In the PICU unit, if there is a blood gas analyzer, how often are they functioning?

◯ Fully functional
◯ Occasionally functional
◯ Not functional

Availability of PICU infrastructure

	Always	Sometimes	Every once in a while	Rarely	Never
Oxygen access	◯	◯	◯	◯	◯
Negative Pressure suction facility	◯	◯	◯	◯	◯
Pediatric ambu bags	◯	◯	◯	◯	◯
Pediatric endo tracheal tubes	◯	◯	◯	◯	◯

Vassopressor drugs for ressuscitation	◯	◯	◯	◯	◯
Pulse Oximetry	◯	◯	◯	◯	◯
Thermometer	◯	◯	◯	◯	◯
Weighing scale	◯	◯	◯	◯	◯
Glucometer	◯	◯	◯	◯	◯
Portable X ray machine	◯	◯	◯	◯	◯
Portable Ultrasound Machine	◯	◯	◯	◯	◯

Pediatric Theatres- Availability of essential equipment and facilities

How many operating theatres for children are there ______________________

How often are the pediatric surgery theatres functional when required
- ◯ Fully functional
- ◯ Occasionally functional
- ◯ Not functional

How many Pediatric Surgery theatres have been requested? ______________________

How many anesthesia machines for children are available ______________________

Availability of Children's Theatre facilities

	Always	Sometimes	Every once in a while	Rarely	Never
Capnography	◯	◯	◯	◯	◯
Arterial blood gas	◯	◯	◯	◯	◯
Oxygen gas	◯	◯	◯	◯	◯
Pulse oximetry	◯	◯	◯	◯	◯
Thermometer	◯	◯	◯	◯	◯
Infusion pump	◯	◯	◯	◯	◯
Bed warmer	◯	◯	◯	◯	◯
Required anasthetic medications	◯	◯	◯	◯	◯

When you consult the following service, how often do you receive it

	Always	Sometimes	Every once in a while	Rarely	Never
Pediatric Neurosurgical service	◯	◯	◯	◯	◯
Pediatric Orthopedic service	◯	◯	◯	◯	◯
Pediatric Urological Service	◯	◯	◯	◯	◯
Pediatric Cardiothoracic Service	◯	◯	◯	◯	◯
Pediatric Dietetics Service	◯	◯	◯	◯	◯

Pediatric Radiological Service	◯	◯	◯	◯	◯
