# Supplementary material for: An assessment of the health system’s capacity for major structural birth defects care in Tanzania using anorectal malformations as a tracer condition: A multi-phase, mixed-methods study protocol
Source: PLoS One. 2026 Oct 5;21(10):e0359639. doi: 10.1371/journal.pone.0359639 (PMC13637857; doi:10.1371/journal.pone.0359639)
Supplement: Supplementary file 4 [file pone.0359639.s004.pdf]

Healthcare worker, preoperative checklist- Practicality and Acceptability Questionnaire

Record ID	__________________________________
Questionnaire Number	__________________________________
Participant Sex	◯ Male<br>◯ Female
Level of Training	◯ Medical Office/ Registrar<br>◯ Junior Surgical resident (< semester 3)<br>◯ Senior surgical resident (>semester 3)<br>◯ General Surgical Specialist<br>◯ Pediatric Surgical trainee<br>◯ Pediatric surgeon
Since your last professional degree, how long have you been in clinical practice (in years)	__________________________________

If I am called to review neonate with anorectal malformation, within 6 hours of request for review, I am

	Strongly Disagree	Disagree	Neither Disagree nor Agree	Agree	Strongly Agree
Capable of fully assessing the neonante	◯	◯	◯	◯	◯
Capable of determining an unstable neonate	◯	◯	◯	◯	◯
I know who to consult for assistance	◯	◯	◯	◯	◯
I can assess for other external malformations	◯	◯	◯	◯	◯
I know which investigations to be ordered	◯	◯	◯	◯	◯

Within the first 48 hours of receiving a neonate with anorectal malformation, how commonly are the following investigations completed?

	Always	Sometimes	Every once in a while	Rarely	Never
ECHO	◯	◯	◯	◯	◯
KUB USS	◯	◯	◯	◯	◯
Babygram/Abdomino pelvic USS	◯	◯	◯	◯	◯

Full blood picture	○	○	○	○	○
Serum Electrolytes	○	○	○	○	○

If the following subspecialised services are required on admission for a neonate with anorectal malformation, how accessible are they within 48 hrs of consultation

	Always	Sometimes	Every once in a while	Rarely	Never
Pediatric Radiology Services	○	○	○	○	○
Pediatric Cardiology Service	○	○	○	○	○
Pediatric Cardiothoracic Services	○	○	○	○	○
Pediatric Nephrology Services	○	○	○	○	○
Pediatric Urology Services	○	○	○	○	○
Pediatric Neurosurgical Services	○	○	○	○	○
Medical Geneticist Services	○	○	○	○	○

Suppose we introduce a checklist, either paper-based, or accessible through your phone- that has the recommendations on what should be done within 6- 48 hours of a neonate with ARM presenting to MNH, in terms of clinical examination to be performed, laboratory and imaging investigations to be conducted, so that the neonate is ready for first surgical intervention

The use of a checklist for evaluation of a neonate with ARM presenting to MNH, will

	Strongly Disagree	Disagree	Neither Disagree nor Agree	Agree	Strongly Agree
Put pressure on my performance	○	○	○	○	○
Help me to remember what must be done	○	○	○	○	○
Increase unnecessary documentation	○	○	○	○	○
Assist in determining areas of improvement in the care	○	○	○	○	○
Assist in determining where I need help	○	○	○	○	○
It's a good idea, but difficult to implement	○	○	○	○	○
It's a good idea, but training of the use is needed	○	○	○	○	○
It's a good idea, but requires a lot of resources and coordination	○	○	○	○	○
It's redundant, this is what we do everyday	○	○	○	○	○

I am keen to try using the checklist and see how it would work	○	○	○	○	○
The checklist will not have an impact on time to first surgical intervention	○	○	○	○	○
The checklist will increase the time to first surgical intervention	○	○	○	○	○
The checklist will decrease time to first surgical intervention	○	○	○	○	○
