# Supplementary material for: An assessment of the health system’s capacity for major structural birth defects care in Tanzania using anorectal malformations as a tracer condition: A multi-phase, mixed-methods study protocol
Source: PLoS One. 2026 Oct 5;21(10):e0359639. doi: 10.1371/journal.pone.0359639 (PMC13637857; doi:10.1371/journal.pone.0359639)
Supplement: Supplementary file 5 [file pone.0359639.s005.docx]

GRAMMS checklist for An Assessment of the Health System's Capacity for Major Structural Birth Defects Care in Tanzania Using Anorectal Malformations: A multi-phase, mixed methods Study protocol.

| **Item** | **Description** | **Where Addressed** |
| --- | --- | --- |
| Justification for using mixed methods | Clear rationale for why a mixed-methods design is needed and how it strengthens the study. | Background (pp. 3–4) — The complexity of birth-defect prevention across tiers of care is detailed, and the integration of quantitative, qualitative, facility-level, and feasibility components is justified. |
| Description of the mixed-methods design | Explicit description of the overall design and its components. | Study Design and Duration (pp. 10–11) — A four-phase mixed-methods design (cross-sectional, phenomenological study, facility survey, feasibility study) is described. |
| Description of each method and sequence | Detailed description of each method, sampling, data collection, and sequencing. | Methods (pp. 11–20) — Phases I–IV are outlined, incorporating sampling frames, procedures, instruments, and sequencing. |
| Integration of methods | How qualitative and quantitative components will be integrated at analysis or interpretation. | Variables section (pp. 18–19) — Variables are mapped to the conceptual framework to illustrate convergence; integration is also reflected in the Discussion. |
| Limitations of the mixed-methods design | Discussion of limitations specific to the design. | Discussion/Conclusion (pp. 31–32) — Limitations are described, including small census sample, single-country context, recall bias, and facility variability. |
| Insights gained from mixing methods | Added value expected from combining methods. | New Evidence paragraph (pp. 4–5) — The combination of prospective assessment, qualitative insights, facility benchmarking, and feasibility evaluation is expected to generate actionable system-level evidence. |
| Transparency of reporting | Clear reporting of procedures, ethics, data management, and analytic transparency. | Data Collection, Data Management, Data Analysis, Ethics (pp. 20–30) — Detailed procedures, analytic plans, ethical approvals, and data handling are provided. |
