# Supplementary material for: An assessment of the health system’s capacity for major structural birth defects care in Tanzania using anorectal malformations as a tracer condition: A multi-phase, mixed-methods study protocol
Source: PLoS One. 2026 Oct 5;21(10):e0359639. doi: 10.1371/journal.pone.0359639 (PMC13637857; doi:10.1371/journal.pone.0359639)
Supplement: Supplementary file 7 [file pone.0359639.s007.docx]

**RESPONSE TO REVIEWER COMMENTS**

We thank the reviewers for their comments aimed at improving the quality of this manuscript. Kindly find our responses to the comments raised below.

**Comment 1:** terminology related to prevention, detection, and management should be used more consistently throughout. please check the consistency through the whole manuscript.

**Response:** We thank the reviewer for this helpful observation. We conducted a comprehensive review of the manuscript and standardized terminology throughout to align with the conceptual framework underpinning the study. Specifically, we consistently describe the continuum as "prevention, early detection, and management" of major structural birth defects. Screening is now presented as a mechanism of detection, while surgical care is described within the broader domain of management. These revisions improve conceptual clarity and consistency across all sections of the manuscript.

**Comment 2:** Justify the multivariable analysis in Phase I, given the relatively small anticipated sample size.

**Response:** We appreciate the reviewer's concern regarding the anticipated sample size. We have revised the Phase I analysis section to provide additional justification for the planned multivariable logistic regression. Because ARM is a rare condition, we acknowledge the limited sample size and the consequent risk of model overfitting. We therefore clarify that (i) only a small number of prespecified clinically relevant predictors will be considered, (ii) the model will only be fitted if the events-per-variable criterion is satisfied, and (iii) if these conditions are not met, analyses will remain descriptive and univariable. We further specify that the multivariable analysis is exploratory and intended to identify potential independent associations for future investigation rather than to develop a definitive predictive model.
