# Supplementary material for: An assessment of the health system’s capacity for major structural birth defects care in Tanzania using anorectal malformations as a tracer condition: A multi-phase, mixed-methods study protocol
Source: PLoS One. 2026 Oct 5;21(10):e0359639. doi: 10.1371/journal.pone.0359639 (PMC13637857; doi:10.1371/journal.pone.0359639)
Supplement: Supplementary file 8 [file pone.0359639.s006.docx]

**Response to reviewer comments**

We thank the reviewers for their comments. Below are the responses to the specific comments

**Reviewer 1 — Major Comment 1**

**Reviewer comment:** *“The recruitment for Phase I of 28 newborn-mother dyads within a one year period seems optimistic… Can recruitment be extended to a 2-year period?”*

**Author response:** Recruitment has now been extended to an **18‑month period**, supported by institutional data showing **15–35 ARM neonatal cases per year**. The sample size has been recalculated using Cochran’s formula with finite population correction, yielding a minimum of **46 participants**, which is feasible within the extended window. Furthermore sampling will be consecutive, for the duration of data collection.

**Reviewer 1 — Major Comment 1a**

**Reviewer comment:** *“Was there a consideration to complete Phase I using retrospective data?”*

**Author response:** We appreciate this suggestion. We elected to retain a **prospective design** because it allows accurate capture of upstream maternal and facility‑level determinants and minimizes recall bias. Retrospective records lack several variables essential to our analysis (e.g., preconception care, antenatal content, birth attendant practices).

**Reviewer 1 — Major Comment 1b**

**Reviewer comment:** *“Sample size may not be large enough for multivariable analysis.”*

**Author response:** The model will use **three prespecified predictors**, ensuring **≥10 events per variable**, and includes full diagnostics (VIF, Box–Tidwell, residuals). This satisfies small‑sample requirements and reduces overfitting risk.

**Reviewer 1 — Major Comment 2**

**Reviewer comment:** *“Please provide a complete list of independent variables for Phase I.”*

**Author response:** We have expanded the Variables section to include **all maternal, periconception, antenatal, delivery, and postnatal variables**, as well as facility‑level characteristics. Phase I data‑collection tool is also shared (Supplementary S1).

**Reviewer 1 — Major Comment 3**

**Reviewer comment:** *“The knowledge gaps/questions that each Phase is meant to address are not clear.”*

**Author response:** We have revised the Background and Conceptual Framework sections to explicitly map each phase to its corresponding knowledge gap. Each phase now has a clearly stated purpose aligned with the study objectives.

**Reviewer 1 — Major Comment 4**

**Reviewer comment:** *“There are no hypotheses stated for the quantitative portions of the study.”*

**Author response:** We have now added explicit hypotheses:

- **Phase I hypothesis:** Early detection of ARM is associated with maternal antenatal uptake, birth attendant practices, and completeness/timing of newborn examination.
- **Phase III hypothesis:** Paediatric surgical capacity will differ significantly across hospitals due to variations in infrastructure, workforce, service delivery, equipment, and governance.

**Reviewer 1 — Major Comment 5**

**Reviewer comment:** *“Please provide more detail about how the qualitative interviews in Phase II will be conducted.”*

**Author response:** We have substantially expanded Phase II methods to include:

- Interviewer identity, training, and positionality
- Reflexivity procedures (bracketing, memoing, nonverbal cue documentation)
- Development and piloting of the interview guide
- Translation and transcript verification
- Detailed analytic steps using the Modified Stevick–Colaizzi–Keen method
- Trustworthiness strategies (credibility, dependability, confirmability, transferability)

Additionally, we explicitly state that **the interviewer (RAB) will not participate in transcript analysis** to minimise bias.

**Reviewer 1 — Major Comment 6**

**Reviewer comment:** *“Phase III is survey-based; please clarify independent variables, respondents, and data collection.”*

**Author response:** We have clarified methods further:

- Respondents (heads of paediatric surgery, NICU, PICU, anaesthesia, and medical director)
- Tool development and contextual adaptation
- Direct observation and inventory verification
- Facility‑level, workforce, service‑delivery, equipment, and governance variables
- Compliance scoring and gap quantification
- Non‑parametric comparative analysis (Friedman, Wilcoxon, chi‑square/Fisher)

**Reviewer 1 — Major Comment 7**

**Reviewer comment:** *“Phase IV is described as an interview with a Likert questionnaire; please clarify.”*

**Author response:** Phase IV data collection is now clearly described as a **researcher‑administered Likert‑type questionnaire**, adapted from validated Acceptability and Feasibility frameworks. We provide details on piloting, domains, reliability testing (Cronbach’s α), and analytic plan.

**Reviewer 1 — Major Comment 8**

**Reviewer comment:** *“How will issues of bias and reflexivity be addressed in Phase II?”*

**Author response:** We have strengthened reflexivity procedures through:

- Bracketing
- Reflexive memoing
- Documentation of nonverbal cues
- Team‑based analysis across diverse disciplinary backgrounds
- Audit trail
- Thick description
- **Explicit statement that the interviewer will not analyse transcripts**

**Reviewer 1 — Minor Comments**

**Minor Comment 1**

**Reviewer comment:** *“Too many abbreviations; some unnecessary.”*

**Author response:** We have removed unnecessary abbreviations (e.g., BD, SSA) and ensured all abbreviations are defined once.

**Minor Comment 2**

**Reviewer comment:** *“Line 159 appears to have an incomplete sentence.”*

**Author response:** This has been corrected.

**Minor Comment 3**

**Reviewer comment:** *“How many paediatric surgeons are in the country?”*

**Author response:** We now state that **60 clinicians**, at MNH pediatric surgery unit (MDs, MMED general surgery trainees, pediatric surgery trainees, General surgeons and pediatric surgeons in the unit) meet eligibility criteria for Phase IV. Published literature states as of 2023 there were 12 pediatric surgeons in Tanzania distributed in Dar es salaam, and the zonal referral hospitals.

**Minor Comment 4**

**Reviewer comment:** *“Typographical errors (e.g., double period).”*

**Author response:** All typographical errors have been corrected.

**Minor Comment 5**

**Reviewer comment:** *“Have all centres agreed to being compared with each other?”*

**Author response:** We have added the explicit statement: “All participating centres reviewed the protocol, which included the comparative benchmarking analysis, and granted institutional approval for participation.”

**Reviewer 2 — Overall Assessment**

**Reviewer comment:** *“Overall, this appears to be a clinically relevant project. However, the manuscript would benefit from improved language, greater consistency regarding the study aims, a more organized description of the methodology, and more detailed explanation of outcome variables. Visual overviews summarizing the study design and project timeline are essential.”*

**Author response:** We thank the reviewer for this constructive summary. The manuscript has undergone comprehensive revision to improve clarity, consistency, and methodological organisation. We have added two visual overviews:

- **Figure 1:** Conceptual framework
- **Figure 2:** Study timeline and phases. Outcome variables have been expanded in each phase, and language throughout has been refined for clarity and readability.

**Reviewer 2 — Comment 1: Abbreviations**

**Reviewer comment:** *“Limit the use of abbreviations to improve readability. Several abbreviations are unnecessary and should be written out in full.”*

**Author response:** We agree and have removed unnecessary abbreviations (e.g., BD, SSA). All remaining abbreviations are now defined once at first use and used sparingly.

**Reviewer 2 — Comment 2: Repeated definitions**

**Reviewer comment:** *“Ensure abbreviations are defined only once. ARM is introduced repeatedly.”*

**Author response:** We have revised the manuscript to ensure ARM and other terms are defined once and used consistently thereafter.

**Reviewer 2 — Abstract Comment 1**

**Reviewer comment:** *“The abstract mentions prevention, yet prevention is not addressed in the proposed study.”*

**Author response:** We have revised the abstract to ensure prevention is described only in relation to Phase I’s upstream determinants and the conceptual framework. The abstract now aligns fully with the study objectives.

**Reviewer 2 — Abstract Comment 2**

**Reviewer comment:** *“Ensure the objectives in the abstract match those in the manuscript.”*

**Author response:** The abstract has been updated to reflect the four study objectives exactly as stated in the main text.

**Reviewer 2 — Background Comment 1**

**Reviewer comment:** *“Reduce abbreviations such as BD and SSA.”*

**Author response:** Completed. These abbreviations have been removed.

**Reviewer 2 — Background Comment 2**

**Reviewer comment:** *“Clarify the statement: ‘There are no ARM-specific primary prevention interventions.’”*

**Author response:** We have clarified this statement and expanded the explanation of generic primary birth defects prevention entry points shared across major structural birth defects.

**Reviewer 2 — Background Comment 3**

**Reviewer comment:** *“The section discussing antenatal care and prevention should be removed or more clearly linked to the aims.”*

**Author response:** We have revised this section to explicitly link antenatal care and preconception care to **Phase I’s upstream determinants** and the conceptual framework.

**Reviewer 2 — Background Comment 4**

**Reviewer comment:** *“Please review the following sentence for language and meaning… It is difficult to understand.”*

**Author response:** The sentence has been rewritten for clarity and coherence.

**Reviewer 2 — Background Comment 5**

**Reviewer comment:** *“Provide a reference supporting the statement that >50% of ARM cases are diagnosed after 48 hours.”*

**Author response:** We have added the appropriate reference (25) and expanded the contextual evidence, including the Tanzanian retrospective review (26).

**Reviewer 2 — Background Comment 6**

**Reviewer comment:** *“Clarify whether you are referring to limited healthcare capacity, lack of trained personnel, or another aspect.”*

**Author response:** We have revised the relevant paragraph to specify gaps in referral pathways, specialised workforce, infrastructure, and in‑hospital processes.

**Reviewer 2 — Background Comment 7**

**Reviewer comment:** *“The manuscript states: ‘This study will benchmark prevention…’ but prevention is not part of the objectives.”*

**Author response:** We have removed this statement and replaced it with a description aligned with the study objectives.

**Reviewer 2 — Objectives Comment**

**Reviewer comment:** *“Objectives are clearly stated, but ensure consistency throughout the manuscript.”*

**Author response:** We have ensured full consistency across the abstract, background, conceptual framework, and methods.

**Reviewer 2 — Methods Comment 1**

**Reviewer comment:** *“Specify the planned study period.”*

**Author response:** The study period is now clearly stated in the Study Design, Setting, and Duration section and illustrated in Figure 2.

**Reviewer 2 — Methods Comment 2**

**Reviewer comment:** *“Provide references for all methods and instruments used.”*

**Author response:** We have added references for all tools, including the NSESSS instrument, Stevick–Colaizzi–Keen method, OReCS checklist, and Acceptability/Feasibility frameworks.

**Reviewer 2 — Methods Comment 3**

**Reviewer comment:** *“Methods section needs more comprehensive description.”*

**Author response:** We have substantially expanded all four phases, including:

- Sampling strategies
- Data‑collection procedures
- Tool development and piloting
- Analytic frameworks
- Trustworthiness strategies
- Facility‑survey procedures
- Feasibility scoring system

**Reviewer 2 — Methods Comment 4**

**Reviewer comment:** *“Include a figure summarizing study phases and a timeline.”*

**Author response:** Completed.

- **Figure 1:** Conceptual framework
- **Figure 2:** Study timeline and phases

**Reviewer 2 — Methods Comment 5**

**Reviewer comment:** *“Language editing by a fluent English speaker is recommended.”*

**Author response:** The manuscript has undergone extensive language revision to improve clarity, coherence, and readability.
