# Supplementary material for: An assessment of the health system’s capacity for major structural birth defects care in Tanzania using anorectal malformations as a tracer condition: A multi-phase, mixed-methods study protocol
Source: PLoS One. 2026 Oct 5;21(10):e0359639. doi: 10.1371/journal.pone.0359639 (PMC13637857; doi:10.1371/journal.pone.0359639)
Supplement: Peer review history

# Peer review: Decision Letter 0

**DOI:** 10.1371/journal.pone.0359639.r001

**Authors:** Susan Horton

7 Jul 2026

Dear Dr. Nyamuryekung'e,

Thank you for submitting your manuscript to PLOS ONE. After careful consideration, we feel that it has merit but does not fully meet PLOS ONE’s publication criteria as it currently stands. Therefore, we invite you to submit a revised version of the manuscript that addresses the points raised during the review process.

Please submit your revised manuscript by Aug 06 2026 11:59PM. If you will need more time than this to complete your revisions, please reply to this message or contact the journal office at plosone@plos.org. When you're ready to submit your revision, log on to https://www.editorialmanager.com/pone/ and select the 'Submissions Needing Revision' folder to locate your manuscript file.

- A letter that responds to each point raised by the academic editor and reviewer(s). You should upload this letter as a separate file labeled 'Response to Reviewers'.
- A marked-up copy of your manuscript that highlights changes made to the original version. You should upload this as a separate file labeled 'Revised Manuscript with Track Changes'.
- An unmarked version of your revised paper without tracked changes. You should upload this as a separate file labeled 'Manuscript'.

If you would like to make changes to your financial disclosure, please include your updated statement in your cover letter. Guidelines for resubmitting your figure files are available below the reviewer comments at the end of this letter.

If applicable, we recommend that you deposit your laboratory protocols in protocols.io to enhance the reproducibility of your results. Protocols.io assigns your protocol its own identifier (DOI) so that it can be cited independently in the future. For instructions see: https://journals.plos.org/plosone/s/submission-guidelines#loc-laboratory-protocols. Additionally, PLOS ONE offers an option for publishing peer-reviewed Lab Protocol articles, which describe protocols hosted on protocols.io. Read more information on sharing protocols at https://plos.org/protocols?utm_medium=editorial-email&utm_source=authorletters&utm_campaign=protocols.

As the corresponding author, your ORCID iD is verified in the submission system and will appear in the published article. PLOS supports the use of ORCID, and we encourage all coauthors to register for an ORCID iD and use it as well. Please encourage your coauthors to verify their ORCID iD within the submission system before final acceptance, as unverified ORCID iDs will not appear in the published article. *Only*  the individual author can complete the verification step; PLOS staff *cannot*  verify ORCID iDs on behalf of authors.

We look forward to receiving your revised manuscript.

Kind regards,

Susan Horton

Academic Editor

PLOS One

Journal Requirements:

When submitting your revision, we need you to address these additional requirements.

1. Please ensure that your manuscript meets PLOS ONE's style requirements, including those for file naming. The PLOS ONE style templates can be found at

https://journals.plos.org/plosone/s/file?id=wjVg/PLOSOne_formatting_sample_main_body.pdf and https://journals.plos.org/plosone/s/file?id=ba62/PLOSOne_formatting_sample_title_authors_affiliations.pdf

2. Please include a complete copy of PLOS’ questionnaire on inclusivity in global research in your revised manuscript. Our policy for research in this area aims to improve transparency in the reporting of research performed outside of researchers’ own country or community. The policy applies to researchers who have travelled to a different country to conduct research, research with Indigenous populations or their lands, and research on cultural artefacts. The questionnaire can also be requested at the journal’s discretion for any other submissions, even if these conditions are not met.  Please find more information on the policy and a link to download a blank copy of the questionnaire here: https://journals.plos.org/plosone/s/best-practices-in-research-reporting. Please upload a completed version of your questionnaire as Supporting Information when you resubmit your manuscript.

3. Your ethics statement should only appear in the Methods section of your manuscript. If your ethics statement is written in any section besides the Methods, please delete it from any other section.

4. Please provide a complete Data Availability Statement in the submission form, ensuring you include all necessary access information or a reason for why you are unable to make your data freely accessible. If your research concerns only data provided within your submission, please write "All data are in the manuscript and/or supporting information files" as your Data Availability Statement.

5. Please include captions for your Supporting Information files at the end of your manuscript, and update any in-text citations to match accordingly. Please see our Supporting Information guidelines for more information: http://journals.plos.org/plosone/s/supporting-information.

6. We notice that your supplementary [files] are included in the manuscript file. Please remove them and upload them with the file type 'Supporting Information'. Please ensure that each Supporting Information file has a legend listed in the manuscript after the references list.

7. If the reviewer comments include a recommendation to cite specific previously published works, please review and evaluate these publications to determine whether they are relevant and should be cited. There is no requirement to cite these works unless the editor has indicated otherwise.

Additional Editor Comments :

The reviewers both agree that this is an important topic. They have both submitted constructive comments designed to strengthen the technical content of the paper, as well as encouraging you to edit the language into the appropriate professional style. I will mention that PLoS does permit you to use e.g. the editor in word processing software or AI, as long as you declare this use (see their website for details).

[Note: HTML markup is below. Please do not edit.]

Reviewers' comments:

Reviewer's Responses to Questions

**Comments to the Author**

1. Does the manuscript provide a valid rationale for the proposed study, with clearly identified and justified research questions?

Reviewer #1: No

Reviewer #2: Yes

**********

2. Is the protocol technically sound and planned in a manner that will lead to a meaningful outcome and allow testing the stated hypotheses?

Reviewer #1: Partly

Reviewer #2: Partly

**********

3. Is the methodology feasible and described in sufficient detail to allow the work to be replicable?

Reviewer #1: No

Reviewer #2: No

**********

4. Have the authors described where all data underlying the findings will be made available when the study is complete?

The PLOS Data policy

Reviewer #1: No

Reviewer #2: No

**********

5. Is the manuscript presented in an intelligible fashion and written in standard English?

Reviewer #1: Yes

Reviewer #2: No

**********

Please use the space provided to explain your answers to the questions above and, if applicable, provide comments about issues authors must address before this protocol can be accepted for publication. You may also include additional comments for the author, including concerns about research or publication ethics.

You may also provide optional suggestions and comments to authors that they might find helpful in planning their study.

Reviewer #1: Thank you for the opportunity to review the article “An Assessment of the Health System's Capacity for Major Structural Birth Defects Care in Tanzania Using Anorectal Malformations, A multi-phase, mixed methods Study protocol.” The stated purpose of this manuscript is to outline a protocol that will describe Tanzania’s capacity to prevent, detect, and manage major structural birth defects. I think this is an interesting, ambitious endeavor with potential very high impact, particularly given that results will be directly shared with hospitals and the Ministry of Health. However, there are aspects of the methods that seem either infeasible or vague, and there are no hypotheses that will guide the analysis for the quantitative aspects of the study.

Major comments:

1. The recruitment for Phase I of 28 newborn-mother dyads within a one year period seems optimistic, when the maximum number of cases is 30 per year. Can recruitment be extended to a 2-year period?

a. Alternatively, was there a consideration to complete the analysis for Phase I using retrospective data (e.g., medical records)? This would potentially resolve (1) any issues with recall bias from patients in the prospective collection of independent variables, and (2) increase the power of Phase I

b. Lines 254-257: I do not think the sample size for Phase I will be large enough to conduct a multivariable analysis. I would remove this sentence if increased enrollment/power is not feasible.

2. Could the authors please provide a complete list of the independent variables for Phase I? It is unclear if clinical variables for the mother (e.g., weight, blood pressure, medications or supplements) or fetus (concomitant birth defects) would be considered for analysis.

3. The knowledge gaps/questions that each Phase is meant to answer/address are not clear.

4. There are no hypotheses stated for the quantitative portions of the study (i.e., Phase I or III).

5. Could the authors provide more detail about how the qualitative interviews in Phase II?

a. Who will participate in the development of the interview guide?

b. Lines 199-202 describe the qualitative analysis using quantitative terms (e.g., independent variables and outcome) yet most qualitative interviews yield exploratory concepts.

c. How is the interviewer (PI) be trained in in their interview style? What is the interviewer’s background/previous experience with this population? Will the interviewer be directly involved in the clinical care of the mother or infant?

d. Will interviewees be compensated for their enrollment?

6. Phase III is survey-based it is unclear how there is an independent variable and outcome with a survey.

a. Please also provide more information on how the survey was developed, and who was involved in its development.

b. I am slightly confused in that Phase III is described as a survey, yet lines 230-232 describe interviews of department heads. Does this mean that the survey will be administered to department heads?

c. There is no information of how survey data will be collected. Will an individual at each hospital observe the systems? Or, will a survey be conducted with operational personnel that manage service delivery, equipment and supplies, etc.?

7. Phase IV is described as “an interview with a pre-tested Likert questionnaire”. Will this phase be an interview, or a survey/Likert questionnaire? Do the authors imply a survey administered by the PI?

a. Please provide a copy of this Likert questionnaire, or at least some more information regarding its development.

8. For Phase II and Phase IV, it appears that only two analysts will be involved in the qualitative analysis, one of whom is the interviewer (PI). How will issues of bias and reflexivity be addressed, since reflexive journaling does not fully account for these issues?

Minor comments:

1. Overall, there are a lot of abbreviations that make the manuscript harder to read. Some examples:

a. Some abbreviations seem unnecessary, such as BD for birth defects

b. Some abbreviations are never defined, such as SSA (line 11), MCHC (line 111)

2. Line 159 appears to have an incomplete sentence that starts with “No exclusions”

3. Phase IV involves assessment of a checklist with pediatric surgical doctors – how many total are in the country? Since recruitment will only occur from hospitals with a pediatric surgeon, presumably every surgeon is eligible for recruitment?

4. Line 202 has two periods at the end of the sentence.

5. Lines 284-286 describe comparisons of compliance scores between facilities. Is this necessary to understand the overall availability of resource to treat ARMs? Have all centers agreed to being compared with each other?

Reviewer #2: Dear authors

Thank you for the opportunity to review this study, which addresses an important and highly relevant topic: improving the care of newborns with anorectal malformations (ARM) in Tanzania. The project has the potential to contribute valuable knowledge in a setting where improved diagnosis and management are greatly needed. Overall, the project appears to be well prepared. However, several aspects of the manuscript require clarification and revision before it can be considered for publication.

Overall assessment: Overall, this appears to be a clinically relevant project. However, the manuscript would benefit from improved language, greater consistency regarding the study aims, a more organized description of the methodology, and more detailed explanation of outcome variables. Importantly: inclusion of visual overviews summarizing the study design and project timeline are essential. I strongly recommend including one or two figures illustrating the overall study design, project phases, and planned workflow. In its current form, the project is difficult to follow, and visual summaries would substantially improve clarity and accessibility.

These revisions would substantially improve the readability and scientific clarity of the manuscript.

In addition: The manuscript would benefit from careful language editing by a fluent English speaker.

In detail:

• I recommend limiting the use of abbreviations to a maximum of two or three throughout the manuscript to improve readability. Several abbreviations are unnecessary (e.g., BD for Birth Defects and SSA for Sub-Saharan Africa) and should preferably be written out in full.

• Please ensure that abbreviations are defined only once. For example, anorectal malformations (ARM) is introduced repeatedly throughout the manuscript.

Abstract

• The abstract mentions prevention, yet prevention is not addressed in the proposed study. Unless prevention is an explicit component of the project, I recommend removing this statement from the abstract.

• Please ensure that the objectives presented in the abstract are fully aligned with those described later in the manuscript.

Background

• Consider reducing the number of abbreviations. For example, Birth Defects does not need to be abbreviated as BD, and Sub-Saharan Africa (SSA) should preferably be written out in full throughout the text.

• Line 14: Please clarify the statement: "There are no ARM-specific primary prevention interventions." Primary prevention of what exactly? Prevention of ARM during pregnancy, prevention through preconception care, or prevention related to congenital anomaly screening? The intended meaning is currently unclear.

• The section discussing antenatal care and prevention of congenital anomalies should either be removed or more clearly linked to the aims of the present project.

• Please review the following sentence for both language and meaning:

"In Tanzania, as in many LMICs in the Maternal and Child Health Care, the preconception care package BD primary prevention are present that can be assessed to establish the current status."

The sentence is difficult to understand and should be rewritten for clarity.

• Line 28: Please provide a reference supporting the statement that in more than 50% of cases, the diagnosis of ARM is made more than 48 hours after birth.

• Lines 29–31: Please clarify whether you are referring to limited healthcare capacity, lack of trained personnel, or another aspect of healthcare delivery. The wording should be revised for clarity.

• Immediately before the Objectives section, the manuscript states: "This study will benchmark prevention..."However, prevention does not appear to be part of the study objectives. Please revise this statement to accurately reflect the scope of the project.

Objectives

• The objectives are clearly stated. However, please ensure that they are presented consistently throughout the manuscript, including in the abstract and that they are consistent also in method.

Methods

• Please specify the planned study period.

• References should be provided for all methods and instruments used in the project.

• For example, please provide the reference for the Severity of Acute Stress Symptoms—Adult (NSESSS)instrument. Has this tool been validated, and has it been validated in the language and cultural context in which it will be used?

• Overall, the methods section would benefit from a more comprehensive description. In particular, I recommend including:

o a figure providing an overview of all study phases and planned methodologies;

o a timeline illustrating the implementation of the entire project.

**********

what does this mean?). If published, this will include your full peer review and any attached files.

If you choose “no”, your identity will remain anonymous but your review may still be made public.

**Do you want your identity to be public for this peer review?**  For information about this choice, including consent withdrawal, please see our Privacy Policy

Reviewer #1: No

Reviewer #2: No

**********

[NOTE: If reviewer comments were submitted as an attachment file, they will be attached to this email and accessible via the submission site. Please log into your account, locate the manuscript record, and check for the action link "View Attachments". If this link does not appear, there are no attachment files.]

To ensure your figures meet our technical requirements, please review our figure guidelines: https://journals.plos.org/plosone/s/figures

You may also use PLOS’s free figure tool, NAAS, to help you prepare publication quality figures: https://journals.plos.org/plosone/s/figures#loc-tools-for-figure-preparation.

NAAS will assess whether your figures meet our technical requirements by comparing each figure against our figure specifications.

# Author comment: Author response to Decision Letter 1

**DOI:** 10.1371/journal.pone.0359639.r002

25 Jul 2026

Response to reviewer comments

We thank the reviewers for their comments. Below are the responses to the specific comments

Reviewer 1 — Major Comment 1

Reviewer comment: “The recruitment for Phase I of 28 newborn-mother dyads within a one year period seems optimistic… Can recruitment be extended to a 2-year period?”

Author response: Recruitment has now been extended to an 18 month period, supported by institutional data showing 15–35 ARM neonatal cases per year. The sample size has been recalculated using Cochran’s formula with finite population correction, yielding a minimum of 46 participants, which is feasible within the extended window. Furthermore sampling will be consecutive, for the duration of data collection.

Reviewer 1 — Major Comment 1a

Reviewer comment: “Was there a consideration to complete Phase I using retrospective data?”

Author response: We appreciate this suggestion. We elected to retain a prospective design because it allows accurate capture of upstream maternal and facility level determinants and minimizes recall bias. Retrospective records lack several variables essential to our analysis (e.g., preconception care, antenatal content, birth attendant practices).

Reviewer 1 — Major Comment 1b

Reviewer comment: “Sample size may not be large enough for multivariable analysis.”

Author response: The model will use three prespecified predictors, ensuring ≥10 events per variable, and includes full diagnostics (VIF, Box–Tidwell, residuals). This satisfies small sample requirements and reduces overfitting risk.

Reviewer 1 — Major Comment 2

Reviewer comment: “Please provide a complete list of independent variables for Phase I.”

Author response: We have expanded the Variables section to include all maternal, periconception, antenatal, delivery, and postnatal variables, as well as facility level characteristics. Phase I data collection tool is also shared (Supplementary S1).

Reviewer 1 — Major Comment 3

Reviewer comment: “The knowledge gaps/questions that each Phase is meant to address are not clear.”

Author response: We have revised the Background and Conceptual Framework sections to explicitly map each phase to its corresponding knowledge gap. Each phase now has a clearly stated purpose aligned with the study objectives.

Reviewer 1 — Major Comment 4

Reviewer comment: “There are no hypotheses stated for the quantitative portions of the study.”

Author response: We have now added explicit hypotheses:

• Phase I hypothesis: Early detection of ARM is associated with maternal antenatal uptake, birth attendant practices, and completeness/timing of newborn examination.

• Phase III hypothesis: Paediatric surgical capacity will differ significantly across hospitals due to variations in infrastructure, workforce, service delivery, equipment, and governance.

Reviewer 1 — Major Comment 5

Reviewer comment: “Please provide more detail about how the qualitative interviews in Phase II will be conducted.”

Author response: We have substantially expanded Phase II methods to include:

• Interviewer identity, training, and positionality

• Reflexivity procedures (bracketing, memoing, nonverbal cue documentation)

• Development and piloting of the interview guide

• Translation and transcript verification

• Detailed analytic steps using the Modified Stevick–Colaizzi–Keen method

• Trustworthiness strategies (credibility, dependability, confirmability, transferability)

Additionally, we explicitly state that the interviewer (RAB) will not participate in transcript analysis to minimise bias.

Reviewer 1 — Major Comment 6

Reviewer comment: “Phase III is survey-based; please clarify independent variables, respondents, and data collection.”

Author response: We have clarified methods further:

• Respondents (heads of paediatric surgery, NICU, PICU, anaesthesia, and medical director)

• Tool development and contextual adaptation

• Direct observation and inventory verification

• Facility level, workforce, service delivery, equipment, and governance variables

• Compliance scoring and gap quantification

• Non parametric comparative analysis (Friedman, Wilcoxon, chi square/Fisher)

Reviewer 1 — Major Comment 7

Reviewer comment: “Phase IV is described as an interview with a Likert questionnaire; please clarify.”

Author response: Phase IV data collection is now clearly described as a researcher administered Likert type questionnaire, adapted from validated Acceptability and Feasibility frameworks. We provide details on piloting, domains, reliability testing (Cronbach’s α), and analytic plan.

Reviewer 1 — Major Comment 8

Reviewer comment: “How will issues of bias and reflexivity be addressed in Phase II?”

Author response: We have strengthened reflexivity procedures through:

• Bracketing

• Reflexive memoing

• Documentation of nonverbal cues

• Team based analysis across diverse disciplinary backgrounds

• Audit trail

• Thick description

• Explicit statement that the interviewer will not analyse transcripts

Reviewer 1 — Minor Comments

Minor Comment 1

Reviewer comment: “Too many abbreviations; some unnecessary.”

Author response: We have removed unnecessary abbreviations (e.g., BD, SSA) and ensured all abbreviations are defined once.

Minor Comment 2

Reviewer comment: “Line 159 appears to have an incomplete sentence.”

Author response: This has been corrected.

Minor Comment 3

Reviewer comment: “How many paediatric surgeons are in the country?”

Author response: We now state that 60 clinicians, at MNH pediatric surgery unit (MDs, MMED general surgery trainees, pediatric surgery trainees, General surgeons and pediatric surgeons in the unit) meet eligibility criteria for Phase IV. Published literature states as of 2023 there were 12 pediatric surgeons in Tanzania distributed in Dar es salaam, and the zonal referral hospitals.

Minor Comment 4

Reviewer comment: “Typographical errors (e.g., double period).”

Author response: All typographical errors have been corrected.

Minor Comment 5

Reviewer comment: “Have all centres agreed to being compared with each other?”

Author response: We have added the explicit statement: “All participating centres reviewed the protocol, which included the comparative benchmarking analysis, and granted institutional approval for participation.”

Reviewer 2 — Overall Assessment

Reviewer comment: “Overall, this appears to be a clinically relevant project. However, the manuscript would benefit from improved language, greater consistency regarding the study aims, a more organized description of the methodology, and more detailed explanation of outcome variables. Visual overviews summarizing the study design and project timeline are essential.”

Author response: We thank the reviewer for this constructive summary. The manuscript has undergone comprehensive revision to improve clarity, consistency, and methodological organisation. We have added two visual overviews:

• Figure 1: Conceptual framework

• Figure 2: Study timeline and phases. Outcome variables have been expanded in each phase, and language throughout has been refined for clarity and readability.

Reviewer 2 — Comment 1: Abbreviations

Reviewer comment: “Limit the use of abbreviations to improve readability. Several abbreviations are unnecessary and should be written out in full.”

Author response: We agree and have removed unnecessary abbreviations (e.g., BD, SSA). All remaining abbreviations are now defined once at first use and used sparingly.

Reviewer 2 — Comment 2: Repeated definitions

Reviewer comment: “Ensure abbreviations are defined only once. ARM is introduced repeatedly.”

Author response: We have revised the manuscript to ensure ARM and other terms are defined once and used consistently thereafter.

Reviewer 2 — Abstract Comment 1

Reviewer comment: “The abstract mentions prevention, yet prevention is not addressed in the proposed study.”

Author response: We have revised the abstract to ensure prevention is described only in relation to Phase I’s upstream determinants and the conceptual framework. The abstract now aligns fully with the study objectives.

Reviewer 2 — Abstract Comment 2

Reviewer comment: “Ensure the objectives in the abstract match those in the manuscript.”

Author response: The abstract has been updated to reflect the four study objectives exactly as stated in the main text.

Reviewer 2 — Background Comment 1

Reviewer comment: “Reduce abbreviations such as BD and SSA.”

Author response: Completed. These abbreviations have been removed.

Reviewer 2 — Background Comment 2

Reviewer comment: “Clarify the statement: ‘There are no ARM-specific primary prevention interventions.’”

Author response: We have clarified this statement and expanded the explanation of generic primary birth defects prevention entry points shared across major structural birth defects.

Reviewer 2 — Background Comment 3

Reviewer comment: “The section discussing antenatal care and prevention should be removed or more clearly linked to the aims.”

Author response: We have revised this section to explicitly link antenatal care and preconception care to Phase I’s upstream determinants and the conceptual framework.

Reviewer 2 — Background Comment 4

Reviewer comment: “Please review the following sentence for language and meaning… It is difficult to understand.”

Author response: The sentence has been rewritten for clarity and coherence.

Reviewer 2 — Background Comment 5

Reviewer comment: “Provide a reference supporting the statement that >50% of ARM cases are diagnosed after 48 hours.”

Author response: We have added the appropriate reference (25) and expanded the contextual evidence, including the Tanzanian retrospective review (26).

Reviewer 2 — Background Comment 6

Reviewer comment: “Clarify whether you are referring to limited healthcare capacity, lack of trained personnel, or another aspect.”

Author response: We have revised the relevant paragraph to specify gaps in referral pathways, specialised workforce, infrastructure, and in hospital processes.

Reviewer 2 — Background Comment 7

Reviewer comment: “The manuscript states: ‘This study will benchmark prevention…’ but prevention is not part of the objectives.”

Author response: We have removed this statement and replaced it with a description aligned with the study objectives.

Reviewer 2 — Objectives Comment

Reviewer comment: “Objectives are clearly stated, but ensure consistency throughout the manuscript.”

Author response: We have ensured full consistency across the abstract, background, conceptual framework, and methods.

Reviewer 2 — Methods Comment 1

Reviewer comment: “Specify the planned study period.”

Author response: The study period is now clearly stated in the Study Design, Setting, and Duration section and illustrated in Figure 2.

Reviewer 2 — Methods Comment 2

Reviewer comment: “Provide references for all methods and instruments used.”

Author response: We have added references for all tools, including the NSESSS instrument, Stevick–Colaizzi–Keen method, OReCS checklist, and Acceptability/Feasibility frameworks.

Reviewer 2 — Methods Comment 3

Reviewer comment: “Methods section needs more comprehensive description.”

Author response: We have substantially expanded all four phases, including:

• Sampling strategies

• Data collection procedures

• Tool development and piloting

• Analytic frameworks

• Trustworthiness strategies

• Facility survey procedures

• Feasibility scoring system

Reviewer 2 — Methods Comment 4

Reviewer comment: “Include a figure summarizing study phases and a timeline.”

Author response: Completed.

• Figure 1: Conceptual framework

• Figure 2: Study timeline and phases

Reviewer 2 — Methods Comment 5

Reviewer comment: “Language editing by a fluent English speaker is recommended.”

Author response: The manuscript has undergone extensive language revision to improve clarity, coherence, and readability.

Attachment Submitted filename: Response to reviewers comments 21.7.26.docx

# Peer review: Decision Letter 1

**DOI:** 10.1371/journal.pone.0359639.r003

**Authors:** Susan Horton

31 Aug 2026

Dear Dr. Nyamuryekung'e,

Thank you for responding to the reviewer comments. Reviewer 2 has 2 remaining minor suggestions to improve exposition. If you can make these changes, I will review your response promptly and issue a final decision.

Please submit your revised manuscript by Sep 30 2026 11:59PM. If you will need more time than this to complete your revisions, please reply to this message or contact the journal office at plosone@plos.org. When you're ready to submit your revision, log on to https://www.editorialmanager.com/pone/ and select the 'Submissions Needing Revision' folder to locate your manuscript file.

Please include the following items when submitting your revised manuscript:

- A letter that responds to each point raised by the academic editor and reviewer(s). You should upload this letter as a separate file labeled 'Response to Reviewers'.
- A marked-up copy of your manuscript that highlights changes made to the original version. You should upload this as a separate file labeled 'Revised Manuscript with Track Changes'.
- An unmarked version of your revised paper without tracked changes. You should upload this as a separate file labeled 'Manuscript'.

If you would like to make changes to your financial disclosure, please include your updated statement in your cover letter. Guidelines for resubmitting your figure files are available below the reviewer comments at the end of this letter.

If applicable, we recommend that you deposit your laboratory protocols in protocols.io to enhance the reproducibility of your results. Protocols.io assigns your protocol its own identifier (DOI) so that it can be cited independently in the future. For instructions see: https://journals.plos.org/plosone/s/submission-guidelines#loc-laboratory-protocols. Additionally, PLOS One offers an option for publishing peer-reviewed Lab Protocol articles, which describe protocols hosted on protocols.io. Read more information on sharing protocols at https://plos.org/protocols?utm_medium=editorial-email&utm_source=authorletters&utm_campaign=protocols.

As the corresponding author, your ORCID iD is verified in the submission system and will appear in the published article. PLOS supports the use of ORCID, and we encourage all coauthors to register for an ORCID iD and use it as well. Please encourage your coauthors to verify their ORCID iD within the submission system before final acceptance, as unverified ORCID iDs will not appear in the published article. *Only*  the individual author can complete the verification step; PLOS staff *cannot*  verify ORCID iDs on behalf of authors.

We look forward to receiving your revised manuscript.

Kind regards,

Susan Horton

Academic Editor

PLOS One

Journal Requirements:

If the reviewer comments include a recommendation to cite specific previously published works, please review and evaluate these publications to determine whether they are relevant and should be cited. There is no requirement to cite these works unless the editor has indicated otherwise.

Please review your reference list to ensure that it is complete and correct. If you have cited papers that have been retracted, please include the rationale for doing so in the manuscript text, or remove these references and replace them with relevant current references. Any changes to the reference list should be mentioned in the rebuttal letter that accompanies your revised manuscript. If you need to cite a retracted article, indicate the article’s retracted status in the References list and also include a citation and full reference for the retraction notice.

Additional Editor Comments:

Thank you for responding to the reviewer comments. Reviewer 2 has made two comments to improve exposition. If you can respond to these comments I will undertake to review the response and make a final decision expeditiously, without needing to resend to reviewers.

[Note: HTML markup is below. Please do not edit.]

Reviewers' comments:

Reviewer's Responses to Questions

**Comments to the Author**

1. Does the manuscript provide a valid rationale for the proposed study, with clearly identified and justified research questions?

Reviewer #1: Yes

Reviewer #2: Yes

**********

2. Is the protocol technically sound and planned in a manner that will lead to a meaningful outcome and allow testing the stated hypotheses?

Reviewer #1: Yes

Reviewer #2: Yes

**********

3. Is the methodology feasible and described in sufficient detail to allow the work to be replicable?

Reviewer #1: Yes

Reviewer #2: Yes

**********

4. Have the authors described where all data underlying the findings will be made available when the study is complete?

The PLOS Data policy

Reviewer #1: Yes

Reviewer #2: Yes

**********

5. Is the manuscript presented in an intelligible fashion and written in standard English?

Reviewer #1: Yes

Reviewer #2: Yes

**********

Please use the space provided to explain your answers to the questions above and, if applicable, provide comments about issues authors must address before this protocol can be accepted for publication. You may also include additional comments for the author, including concerns about research or publication ethics.

You may also provide optional suggestions and comments to authors that they might find helpful in planning their study.

Reviewer #1: I have no further comments and my previous comments were appropriately addressed. I look forward to seeing the results of this important work.

Reviewer #2: The study is now overall well justified. A particular strength is its health-system-wide perspective, using anorectal malformation as a tracer condition across multiple levels of care.

The manuscript would nevertheless benefit from some further minor refinements. In particular, 1. terminology related to prevention, detection, and management should be used more consistently throughout. please check the consistency through the whole manuscript. Also, 2. please justify the multivariable analysis in Phase I, given the relatively small anticipated sample size.

**********

what does this mean?). If published, this will include your full peer review and any attached files.

If you choose “no”, your identity will remain anonymous but your review may still be made public.

**Do you want your identity to be public for this peer review?**  For information about this choice, including consent withdrawal, please see our Privacy Policy

Reviewer #1: **Yes:**  Joyce L. Woo

Reviewer #2: **Yes:**  Pernilla Stenström

**********

[NOTE: If reviewer comments were submitted as an attachment file, they will be attached to this email and accessible via the submission site. Please log into your account, locate the manuscript record, and check for the action link "View Attachments". If this link does not appear, there are no attachment files.]

To ensure your figures meet our technical requirements, please review our figure guidelines: https://journals.plos.org/plosone/s/figures

You may also use PLOS’s free figure tool, NAAS, to help you prepare publication quality figures: https://journals.plos.org/plosone/s/figures#loc-tools-for-figure-preparation.

NAAS will assess whether your figures meet our technical requirements by comparing each figure against our figure specifications.

# Author comment: Author response to Decision Letter 2

**DOI:** 10.1371/journal.pone.0359639.r004

11 Sep 2026

RESPONSE TO REVIEWER COMMENTS

We thank the reviewers for their comments aimed at improving the quality of this manuscript. Kindly find our responses to the comments raised below.

Comment 1: terminology related to prevention, detection, and management should be used more consistently throughout. please check the consistency through the whole manuscript.

Response: We thank the reviewer for this helpful observation. We conducted a comprehensive review of the manuscript and standardized terminology throughout to align with the conceptual framework underpinning the study. Specifically, we consistently describe the continuum as "prevention, early detection, and management" of major structural birth defects. Screening is now presented as a mechanism of detection, while surgical care is described within the broader domain of management. These revisions improve conceptual clarity and consistency across all sections of the manuscript.

Comment 2: Justify the multivariable analysis in Phase I, given the relatively small anticipated sample size.

Response: We appreciate the reviewer's concern regarding the anticipated sample size. We have revised the Phase I analysis section to provide additional justification for the planned multivariable logistic regression. Because ARM is a rare condition, we acknowledge the limited sample size and the consequent risk of model overfitting. We therefore clarify that (i) only a small number of prespecified clinically relevant predictors will be considered, (ii) the model will only be fitted if the events-per-variable criterion is satisfied, and (iii) if these conditions are not met, analyses will remain descriptive and univariable. We further specify that the multivariable analysis is exploratory and intended to identify potential independent associations for future investigation rather than to develop a definitive predictive model.

Attachment Submitted filename: Response to reviewer comments 09.09.26.docx

# Editor report: Decision Letter 2

**DOI:** 10.1371/journal.pone.0359639.r005

**Authors:** Susan Horton

16 Sep 2026

An Assessment of the Health System's Capacity for Major Structural Birth Defects Care in Tanzania Using Anorectal Malformations as a Tracer Condition: A Multi-Phase, Mixed-Methods Study Protocol.

PONE-D-26-23686R2

Dear Dr. Nyamuryekung'e,

We’re pleased to inform you that your manuscript has been judged scientifically suitable for publication and will be formally accepted for publication once it meets all outstanding technical requirements.

Within one week, you’ll receive an e-mail detailing the required amendments. When these have been addressed, you’ll receive a formal acceptance letter and your manuscript will be scheduled for publication.

An invoice will be generated when your article is formally accepted. Please note, if your institution has a publishing partnership with PLOS and your article meets the relevant criteria, all or part of your publication costs will be covered. Please make sure your user information is up-to-date by logging into Editorial Manager at Editorial Manager® and clicking the ‘Update My Information' link at the top of the page. For questions related to billing, please contact billing support.

If your institution or institutions have a press office, please notify them about your upcoming paper to help maximize its impact. If they’ll be preparing press materials, please inform our press team as soon as possible -- no later than 48 hours after receiving the formal acceptance. Your manuscript will remain under strict press embargo until 2 pm Eastern Time on the date of publication. For more information, please contact onepress@plos.org.

Kind regards,

Susan Horton

Academic Editor

PLOS One

Additional Editor Comments (optional):

Reviewers' comments:

# Editor report: Acceptance letter

**DOI:** 10.1371/journal.pone.0359639.r006

**Authors:** Susan Horton

PONE-D-26-23686R2

PLOS One

Dear Dr. Nyamuryekung'e,

I'm pleased to inform you that your manuscript has been deemed suitable for publication in PLOS One. Congratulations! Your manuscript is now being handed over to our production team.

At this stage, our production department will prepare your paper for publication. This includes ensuring the following:

* All references, tables, and figures are properly cited

* All relevant supporting information is included in the manuscript submission,

* There are no issues that prevent the paper from being properly typeset

You will receive further instructions from the production team, including instructions on how to review your proof when it is ready. Please keep in mind that we are working through a large volume of accepted articles, so please give us a few days to review your paper and let you know the next and final steps.

Lastly, if your institution or institutions have a press office, please let them know about your upcoming paper now to help maximize its impact. If they'll be preparing press materials, please inform our press team within the next 48 hours. Your manuscript will remain under strict press embargo until 2 pm Eastern Time on the date of publication. For more information, please contact onepress@plos.org.

You will receive an invoice from PLOS for your publication fee after your manuscript has reached the completed accept phase. If you receive an email requesting payment before acceptance or for any other service, this may be a phishing scheme. Learn how to identify phishing emails and protect your accounts at https://explore.plos.org/phishing.

If we can help with anything else, please email us at customercare@plos.org.

Thank you for submitting your work to PLOS One and supporting open access.

Kind regards,

PLOS One Editorial Office Staff

on behalf of

Dr. Susan Horton

Academic Editor

PLOS One
